# Comprehensive metabolic and transcriptomic profiling of various tissues provide insights for saponin biosynthesis in the medicinally important *Asparagus racemosus*

**DOI:** 10.1038/s41598-018-27440-y

**Published:** 2018-06-14

**Authors:** Prabhakar Lal Srivastava, Anurag Shukla, Raviraj M. Kalunke

**Affiliations:** 1 Symbiosis School of Biological Sciences, Symbiosis International (Deemed University), Lavale, Pune, 412115 India; 2 Biochemical Sciences Division, CSIR-National Chemical Laboratory, Dr. Homi Bhabha Road, Pashan, Pune, 411008 India

## Abstract

*Asparagus racemosus* (Shatavari), belongs to the family *Asparagaceae* and is known as a “curer of hundred diseases” since ancient time. This plant has been exploited as a food supplement to enhance immune system and regarded as a highly valued medicinal plant in Ayurvedic medicine system for the treatment of various ailments such as gastric ulcers, dyspepsia, cardiovascular diseases, neurodegenerative diseases, cancer, as a galactogogue and against several other diseases. In depth metabolic fingerprinting of various parts of the plant led to the identification of 13 monoterpenoids exclusively present in roots. LC-MS profiling led to the identification of a significant number of steroidal saponins (33). However, we have also identified 16 triterpene saponins for the first time in *A. racemosus*. In order to understand the molecular basis of biosynthesis of major components, transcriptome sequencing from three different tissues (root, leaf and fruit) was carried out. Functional annotation of *A. racemosus* transcriptome resulted in the identification of 153 transcripts involved in steroidal saponin biosynthesis, 45 transcripts in triterpene saponin biosynthesis, 44 transcripts in monoterpenoid biosynthesis and 79 transcripts in flavonoid biosynthesis. These findings will pave the way for better understanding of the molecular basis of steroidal saponin, triterpene saponin, monoterpenoids and flavonoid biosynthesis in *A. racemosus*.

## Introduction

*Asparagus racemosus* is one of the most valuable medicinal plants, regarded as a “Queen of herbs” in Ayurvedic health system and has been used worldwide to cure various diseases^1^. It is commonly known as Shatavari, Satawar or Satmuli and belongs to the *Asparagaceae* family (monocot). The plant grows in tropical and temperate deciduous forests and is widely distributed all over India, Africa, Australia, Nepal and Sri Lanka^1^. The genus *Asparagus* comprises of almost 300 species among which *A. racemosus* is the most important species owing to its medicinal properties against various ailments such as, gastric ulcers, dyspepsia, cardiovascular diseases, neurodegenerative disorders, cancer, as a galactogogue and against several other diseases^2,3^. The tuberous root extracts of *A. racemosus* as well as dried root powder have been shown to possess numerous biological activities such as antioxidant, immunomodulatory, anti-inflammatory, anti-bronchitis, anti-dyspepsia and potential broad spectrum antibiotic properties^3,4^. Due to its broad range of medical applications, this plant is recognized as an aphrodisiac, antispasmodic, diuretic, galactogogue and refrigerant since Ayurvedic health system. Owing to its varied applications against several diseases, demand for this plant is continuously increasing. Overharvesting and deforestation are accountable for the consumption of large amounts of natural resources of this highly valuable medicinal plant.

Dried root extracts of *A. racemosus* are known to contain several steroidal saponins known as shatavarins and aglycon portion of saponin: sarsasapogenin^4–7^ with Shatavarin I, IV and V as the major components. Flowers and fruits of *A. racemosus* are known to contain flavonoids such as quercetin and its glycosides, rutin (quercetin 3-O-rutinoside) and hyperoside (quercetin 3-O-galactoside), whereas quercetin 3-glucuronide, ferulic, caffeic and chlorogenic acids have been isolated from the leaf extracts^3,4^. The supply of these natural products suffers from low yields, impurities and consumption of large amounts of natural resources^8^. Chemical synthesis of these natural products is difficult and costly because of their structural complexity. Engineering of metabolic pathway in heterologous systems for the production of terpenoids in large scale presents a cost-effective alternative tool for isoprenoid biosynthesis^9–12^. In plants, RNA sequencing has emerged as a powerful technique for the profiling of complete coding sequences to discover novel genes or gene families, transcription factors involved in various uncharacterized biosynthetic pathways, differential gene expression and tissue specific gene expression patterns^13–16^.

Although recent studies report the transcriptome analysis from *A. racemosus*^17^, no efforts have been made towards in-depth metabolic profiling using GC-MS/LC-MS platform in combination with comprehensive transcriptomic analysis from various tissues. Due to diversified therapeutic applications of *A. racemosus*, it is of great interest to perform the comprehensive metabolic and transcriptome profiling to have better insight about metabolic content and the molecular basis of their biosynthesis. In this manuscript, we present the metabolic fingerprinting and transcriptome sequencing of three different tissues (root, leaf and fruit) of *A. racemosus* to understand the molecular basis for the biosynthesis of steroidal saponins, triterpene saponins, monoterpenoids and flavonoids. The data presented in this manuscript provides a comprehensive resource to metabolic and transcriptomic profile of *A. racemosus* and insights into the biosynthesis of their major active components. Understanding the biosynthetic pathway of shatavarins will pave the way for large-scale production of these highly valued compounds using metabolic engineering tools.

## Results and Discussion

### Metabolic fingerprinting

**Figure 1:**
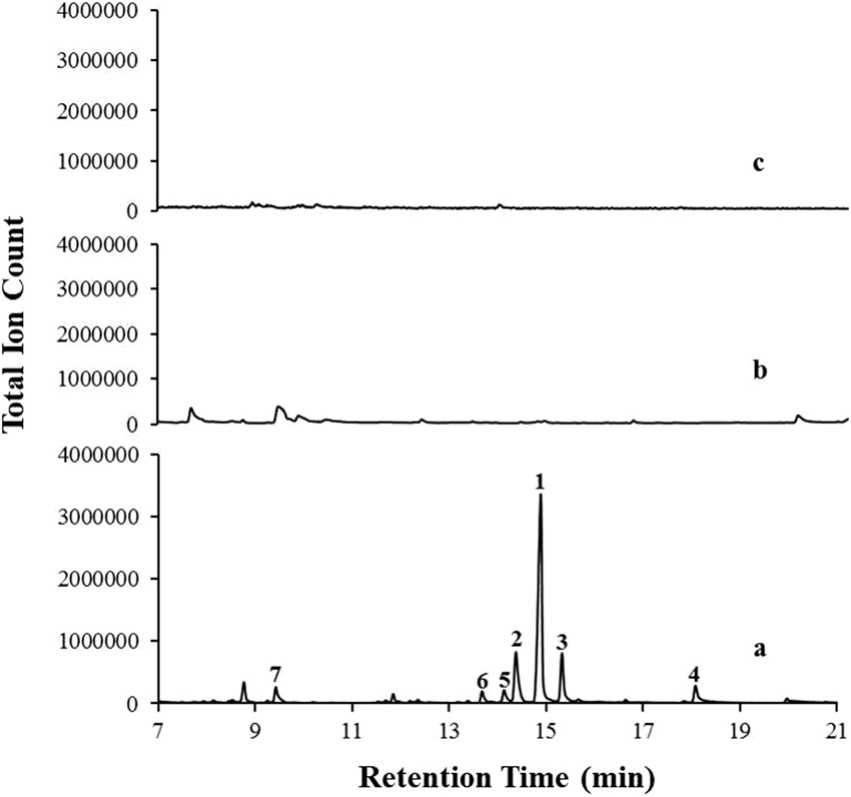
GC-MS profile of n-hexane extract of various tissues from *A. racemosus*. (**a)** Root extract. (**b)** Leaf extract. (**c)** Fruit extract.

A metabolic profile of the various tissues of the plant would give a clear picture of the underlying cellular processes. In order to get a brief overview of the metabolite content present in *A. racemosus*, a systematic metabolic profiling of various tissues (root, leaf and fruit) was carried out. Analysis of non-polar fractions (n-hexane extracts) by GC-MS resulted in the identification of thirteen terpenoids (monoterpenoids) exclusively present in the root tissues (Fig. 1). However, no terpenes were detected in the hexane extracts of leaf or fruit tissues. The major metabolites were identified as: L-*trans*-pinocarveol (**6**, 2.97%), d-verbenol (**5**, 3.53%), (S)-*cis*-verbenol (**2**, 14.53%), (−)-borneol (**1**, 59.20%), 2-pinen-10-ol (**3**, 11.59%), 5-caranol (**4**, 5.19%) along with traces of limonene, pinene, camphene-6-ol, ocimene and teresantalol. Metabolites were identified based on comparison of the mass fragmentation pattern with NIST/Wiley mass spectral library search (Figs S1–S13). This is the first report that shows presence of these monoterpenoids in *A. racemosus* root.

**Figure 2:**
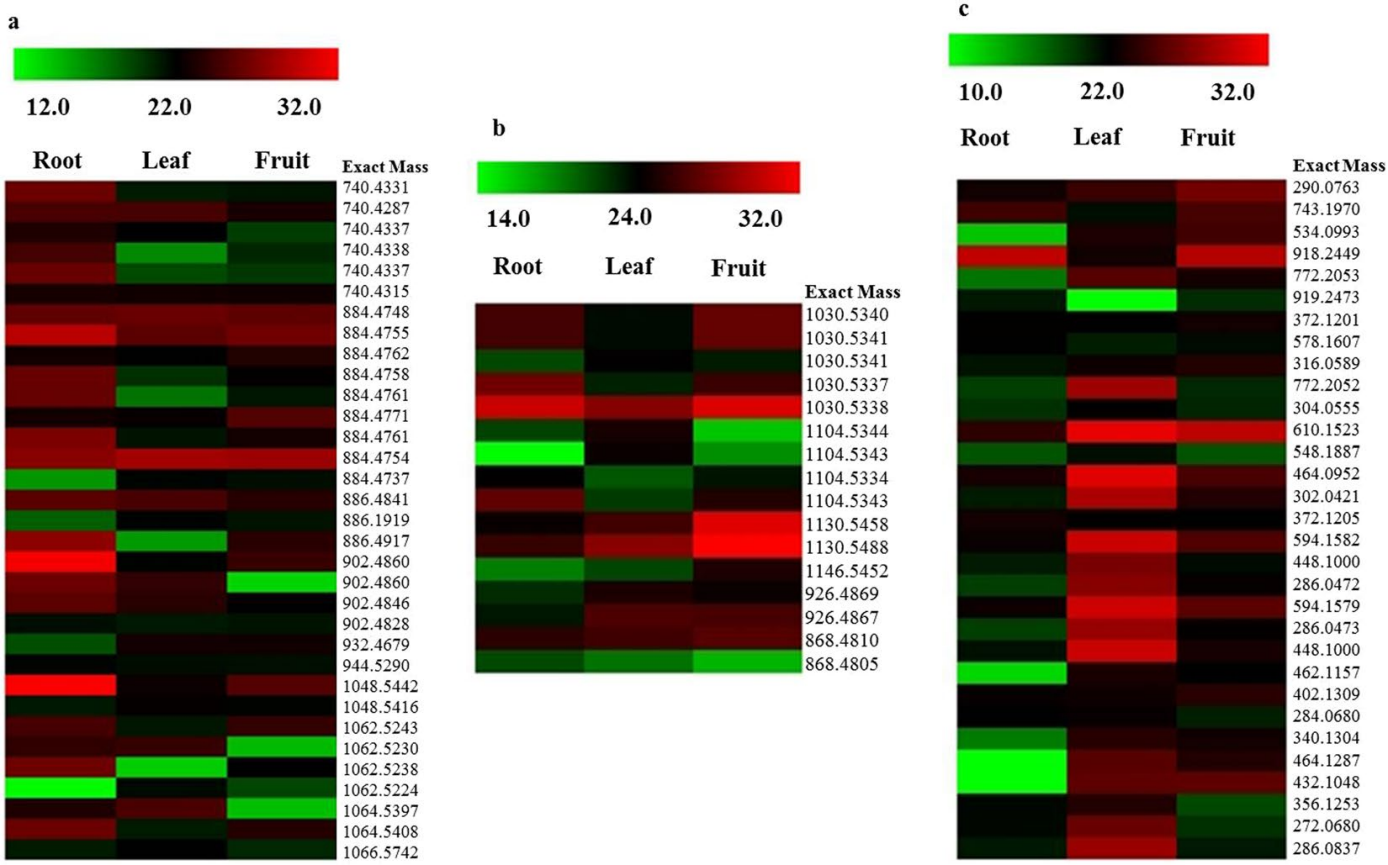
Heatmap depicting the putatively identified saponins and flavonoids present in various tissues of *A. racemosus*. (**a)** Steroidal saponins. (**b)** Triterpene saponins. (**c)** Flavonoids. Color scale showing normalized abundance values of metabolites is shown at the top.

*A. racemosus* is known to contain steroidal saponins, however, a limited number of steroidal saponins have been reported from the root and leaf with Shatavarin I and IV as major metabolites. The LC-MS profile of 75% methanolic extracts (Fig. S14A–C) revealed the presence of a large number of metabolites belonging to steroidal saponins, triterpene saponins and flavonoids with substantial differences in their relative abundance in different tissues (root, leaf and fruit) (Fig. 2). The high sensitivity of the LC-MS has enabled us to identify 33 steroidal saponins suggesting that the number of steroidal saponins detected in *A. racemosus* is much higher than in earlier reports. The MS analysis was performed in order to characterize the saponins detected in the different tissues of *A. racemosus* by comparing the exact observed mass with those reported in KEGG database (Table S1). Our metabolic profiling is in well agreement with earlier reports revealing Shatavarin IV and V as a major metabolites^5,6,18^.

Apart from steroidal saponins, we have also identified 16 triterpene saponins, which are differentially present in *A. racemosus*. So far no triterpene saponins have been reported from this plant to the best of our knowledge. Triterpene saponins were identified by comparing the exact observed mass with the KEGG database (Table S1). It was observed that *A. racemosus* also contains numerous flavonoids. Upon screening of our LC-MS profile for the presence of flavonoids in all the three tissues, a total of 31 flavonoids were detected based on their match with the KEGG database (Table S1). The metabolic profile of flavonoids was found to be dominant in leaf and fruit tissues as compared to the root (Fig. 2c). From the LC-MS profile, it is clear that steroidal saponins are dominant in root as compared to other tissues, whereas triterpene saponins are dominated in fruit and leaf tissues as compared to the root. Further study needs to be carried out for the structural characterization of saponins detected in LC-MS. In order to understand the molecular basis for the biosynthesis of differentially distributed secondary metabolites detected by GC-MS and LC-MS, deep transcriptome sequencing from all the three tissues was carried out in two biological replicates.

### RNA sequencing and *de novo* transcriptome assembly

RNA-seq analysis provides an excellent opportunity to generate comprehensive sequence resources of coding sequences of the genome without whole genome sequencing^16,19^. In order to generate the transcriptome of *A. racemosus*, the high quality total RNA isolated from various tissues was subjected to sequencing on Illumina platform in two biological replicates. A total of 435,463,496 raw reads ranging from 63 to 87 million for each sample and 408,372,236 (93.77%) high quality reads ranging from 59 to 82 million for each sample were generated with a length of 100 bp (Table 1). Additionally, the preprocessed reads were classified into nuclear read (261,300,982) and organelle read (104,016,180) by comparing with the manually curated database (Table 2). Furthermore, the high quality nuclear reads were used for transcriptome assembly for functional annotation and understanding the molecular basis of saponin biosynthesis. The transcriptome assembly and functional annotation of organelle reads were carried out separately, which resulted in the identification of organelle-specific genes (data not shown).

**Table 1:** Summary of raw reads, B1: Biological replicate 1, B2: Biological replicate 2.

Sample Name	No of raw reads	Q_30	High Quality reads	Read length
Root-B1	69,442,146	93.465	64904102	100 × 2
Root-B2	67,382,926	93.045	62696443	100 × 2
Leaf-B1	87,560,292	93.86	82184090	100 × 2
Leaf-B2	70,944,252	94.09	66751447	100 × 2
Fruit-B1	76,877,380	94.09	72333927	100 × 2
Fruit-B2	63,256,500	94.065	59502227	100 × 2

**Table 2:** Summary of Nuclear and Organelle filtered reads and mapping on transcriptome.

	Nuclear raw reads						Organelle reads					
	Root-B1	Root-B2	Leaf-B1	Leaf-B2	Fruit-B1	Fruit-B2	Root-B1	Root-B2	Leaf-B1	Leaf-B2	Fruit-B1	Fruit-B2
No. of filtered reads	51,182,470	50,284,040	31,073,232	26,484,038	54,568,358	47,708,844	6,498,474	5,099,940	43,284,538	34,270,502	10,196,270	4,666,456
Number of reads aligned back to the assembly	48,254,832	47,774,866	27,428,342	24,243,488	49,198,832	43,710,842	6,457,534	5,065,260	43,210,954	34,212,242	10,155,484	4,652,456
Alignment %	94.28	95.01	88.27	91.54	90.16	91.62	99.37	99.32	99.83	99.83	99.6	99.7

**Figure 3:**
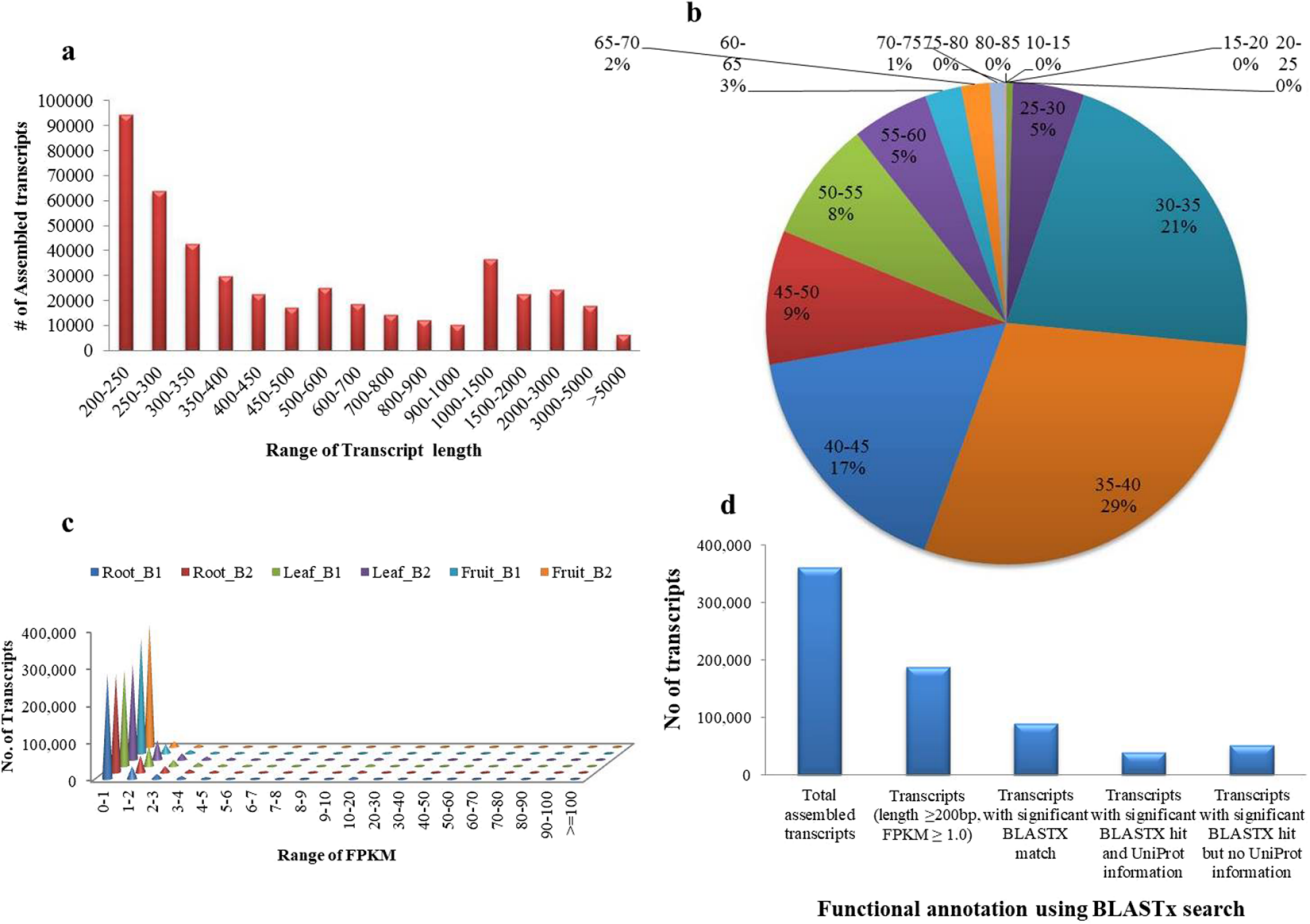
Transcriptome analysis of *A. racemosus*. (**a)** Transcript length distribution of assembled transcripts. (**b)** GC percentage distribution. (**c)** Expression value (FPKM) distribution of assembled transcripts in each tissue. (**d)** Functional annotation of assembled transcripts using BLASTx search.

*De novo* transcriptome assembly of high-quality nuclear reads were performed employing three different softwares (Trinity, Velvet-Oases, and SOAPdenovo) with default parameters. A comparative analysis of the assemblies obtained from all the three tools was performed based on various parameters such as average contig length, N50 contig length and number of contigs generated. Based on assembly statistics, Velvet-Oases was found to be best among all the three assemblers employed in this study, which generated 206,004 transcripts with largest average contig length (1212 bp) and N50 length (2351 bp) followed by Trinity generating 362213 transcripts with average contig length of 779 bp and N50 length of 1420 bp with default parameters for nuclear reads (Table S2). A total of 74221 (36%) transcripts were found to be of more than 1 kb size in velvet-oases assembly. However, 74878 (20.67%) transcripts were found to be more than 1 kb in Trinity assembly. In order to select the best assembly for further analysis, we aligned back trimmed reads to the assembly generated from three different assemblers using Bowtie2 program. Of all filtered reads, about ~91.81% of reads from each sample were accurately aligned back to the assembled transcriptome generated from Trinity, whereas very less percentage of reads were aligned back with the assembly generated using Velvet-Oases and SOAPDenovo (Table S3). Based on this initial information, we selected the transcriptome generated using Trinity for all further analysis. The unique transcripts obtained after *de novo* assembly were designated as *Asparagus racemosus* tentative consensus (ArTc) transcripts and assigned with unique identifier number from ArTc0000001 to ArTc0362213. The total size of the assembly was found to be ~282 Mb with 20.67% (74221) of transcripts larger than 1 kb size (Fig. 3a). The average GC content of *Asparagus racemosus* transcriptome generated from Trinity assembler was found to be 39.81% after CD-HIT test. A total of 242605 (66.97%) transcripts were found to have GC content between 30–45%, however, 80855 (22.32%) transcripts were found to have GC content between 45–60% (Fig. 3b). Transcript expression distribution (FPKM) of all the samples was calculated (Fig. 3c) and we selected the 188,243 transcripts having length ≥ 200 bp and FPKM value ≥ 1 for functional annotation and further analysis.

### Functional annotation

**Figure 4:**
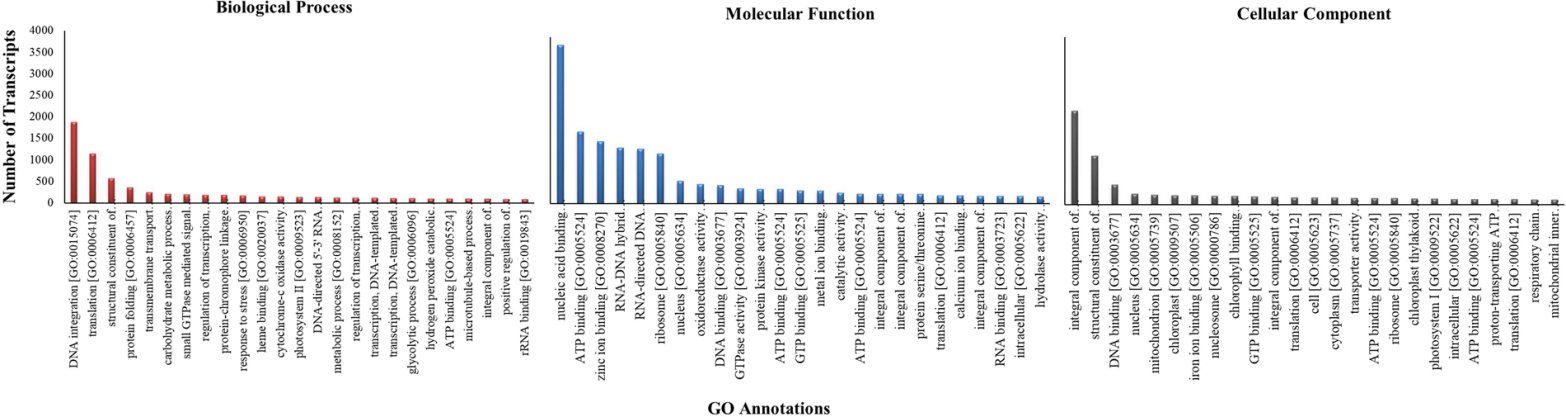
GO annotation of *A. racemosus* transcriptome under biological process, molecular function and cellular component categories.

Considering the complexity in estimating the number of genes and assigning the putative functions to transcripts due to lack of reference genome, functional annotation of *A. racemosus* assembled transcripts was performed based on similarity search with proteins/transcripts in the publically available databases. A total of 188,243 transcripts were used for functional annotation, which yielded the 89,804 (47.77%) of functionally annotated transcripts with confidence (E-value ≤ 1E-05 and identity cutoff of 40%), whereas rest of the transcripts are considered probably specific to *A. racemosus* whose function is not yet assigned. A total of 38755 transcripts were annotated with significant BLASTx hits with Uniprot information, whereas 51049 transcripts were annotated with significant BLASTx hits without Uniprot information (Fig. 3d). We further assigned the gene ontology (GO) terms for the annotated transcripts under biological process, molecular function and cellular components categories. A total of 2712 terms were assigned to 20161 (22.45%) transcripts in biological process category, whereas 3200 terms were assigned for 41369 (46.05%) transcripts in molecular function category. 1816 terms were assigned for 18920 (21.06%) transcripts in case of cellular component category. Top 25 categories annotated in biological processes, molecular function and cellular components are depicted in Fig. 4. DNA integration (1875), translation (1146), structural constituent of ribosome (567), protein folding (355), and transmembrane transport (250) represent the largest class of transcripts annotated under biological processes. However, in case of cellular component categories, top five classes are represented by integral component of membrane (2237), structural constituent of ribosome (1166), DNA binding (474), nucleus (259) and mitochondrion (238). Top five categories of transcripts annotated under molecular functions are represented by: nucleic acid binding (3808), ATP binding (1734), zinc ion binding (1498), RNA-DNA hybrid ribonuclease activity (1350) and RNA directed DNA polymerase activity (1325). We also performed the similarity search of *A. racemosus* transcripts with the available transcriptomics/genomics resource of plants reported till date. A maximum of 19544 (21.76%) transcripts were found to match significantly with *Elaeis guineensis* followed by 14260 (15.87%) transcripts with *Phoenix dactylifera*, 5881 (6.5%) transcripts with *Musa acuminate*, 4832 (5.3%) transcripts with *Asparagus officinalis* and 4246 (4.7%) transcripts with *Ananas comosus* (Fig. S15).

In order to assign KEGG Orthology (KO) number and determine biosynthetic pathways represented in transcriptome, *A. racemosus* transcriptome were compared with manually curated KEGG (Kyoto Encyclopedia of Genes and Genomes) database using BLASTx. A total of 41827 (11.54%) transcripts were assigned with KO numbers representing 375 different KEGG metabolic pathways involved in majority of plant biochemical pathways such as metabolism, cellular processes and genetic information processing (Fig. S16). Out of 41827 transcripts 21691 transcripts were found to be involved in metabolism. A total of 542 transcripts were found to be specifically involved in secondary metabolic pathways such as terpenoid backbone pathway, monoterpenoid biosynthesis, steroid biosynthesis, flavonoid biosynthesis and alkaloid biosynthesis (Fig. S17). These annotations provide a valuable insight for investigating the molecular basis of specific function and diverse pathways in *A. racemosus*.

### Identification of SSRs and transcription factors

**Figure 5:**
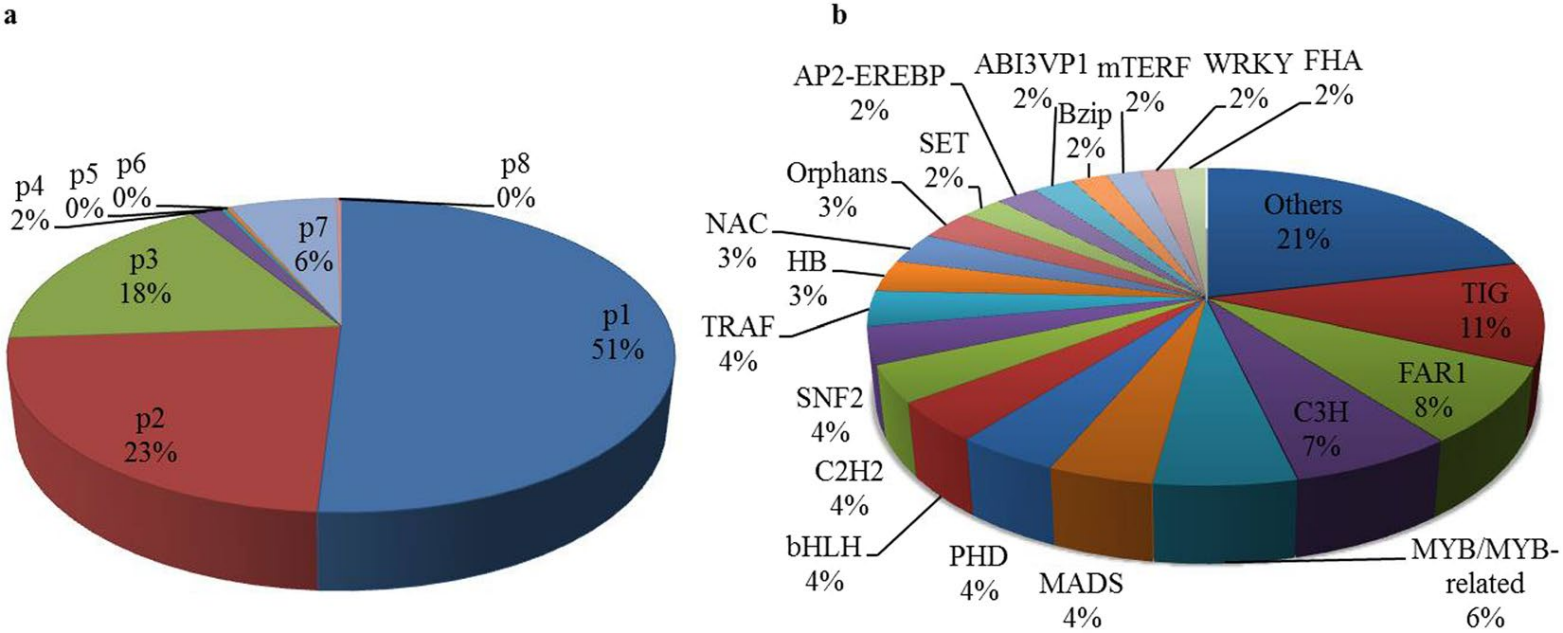
Identification of simple sequence repeats (SSRs) and transcription factors (TFs) encoding transcripts in *A. racemosus* transcriptome. (**a**) SSRs distribution. (**b**) Transcription factors (TFs) distribution in *A. racemosus* transcriptome.

SSRs (microsatellites) have been widely used as molecular markers for determining genetic variations among the different species due to their abundance, ease of development and reproducibility^20^. A total of 40837 SSRs with a minimum length of 10 bp were predicted from 188,243 unique contigs belonging to 8 classes of microsatellites with frequency of one SSR per 6.9 kb of transcript. The identified SSRs were dominated by mono nucleotide (p1), di-nucleotide (p2), tri-nucleotide (p3) and complex repeats (c) representing about 50.93% (20800), 22.91% (9356), 17.37% (7096) and 6.21% (2538), respectively, of the total SSRs (Fig. 5a). In case of mononucleotide repeats, the abundance of A/T repeat showed highest frequency (19755, 94.97%) as compared to G/C repeats (1045, 5.02%). Among the di-nucleotide repeats, AG/CT (3061, 32.71%), GA/TC (2933, 31.34%), TG/CA (1065, 11.38%) and AC/GT (899, 9.6%) showed highest frequency. However, in case of tri-nucleotide repeats, AAT/ATT (593, 8.35%), GAG/CTC (575, 8.1%), TTA/ATT (517, 7.2%) and TTC/GAA (463 6.5%) showed highest occurrence along with GTA/TAC (13) and AGT/ACT (12) being the least abundant. A very less number of SSRs represented by tetra-nucleotide (p4), 1.6% (689), penta-nucleotide (p5), 0.3% (138), hexa-nucleotide (p6), 0.27% (111) and more complex repeats (c*), 0.26% (109) repeats were also identified in the *A. racemosus* transcriptome (Fig. 5a). The discovery of such a large number of SSRs in *A. racemosus* transcriptome would significantly enhance genotyping studies for numerous applications.

*A. racemosus* transcriptome was also scanned for the transcripts encoding transcription factors (TFs). Transcription factors are known to be represented by diverse multi-gene families and play a key role in controlling the expression of single or multiple genes through sequence specific binding to the *cis*-acting element in the promoter regions of target genes^21,22^. We have identified 11758 (3.24%) putative transcription factor encoding transcripts belonging to 83 different families in the *A. racemosus* transcriptome having at least 40% identity with reported databases. The largest members of transcription factors detected in *A. racemosus* are TIG (1241, 10.55%) followed by FAR1 (925, 7.86%), C3H (785, 6.67%), MYB/MYB related (680, 5.78%), MADS (500, 4.25%), PHD (493, 4.19%), bHLH (476, 4.04%), C2H2 (444, 3.77%), SNF (436, 3.70%) and TRAF (426, 3.62%) (Fig. 5b**)**. Several members of TF families, such as AP2-EREBP, bHLH, C2C2, MYB/MYB related and WRKY are reported to regulate secondary metabolism biosynthesis in plants^23–25^ were found to be significantly present in the *A. racemosus* transcriptome.

### Differential gene expression analysis

**Figure 6:**
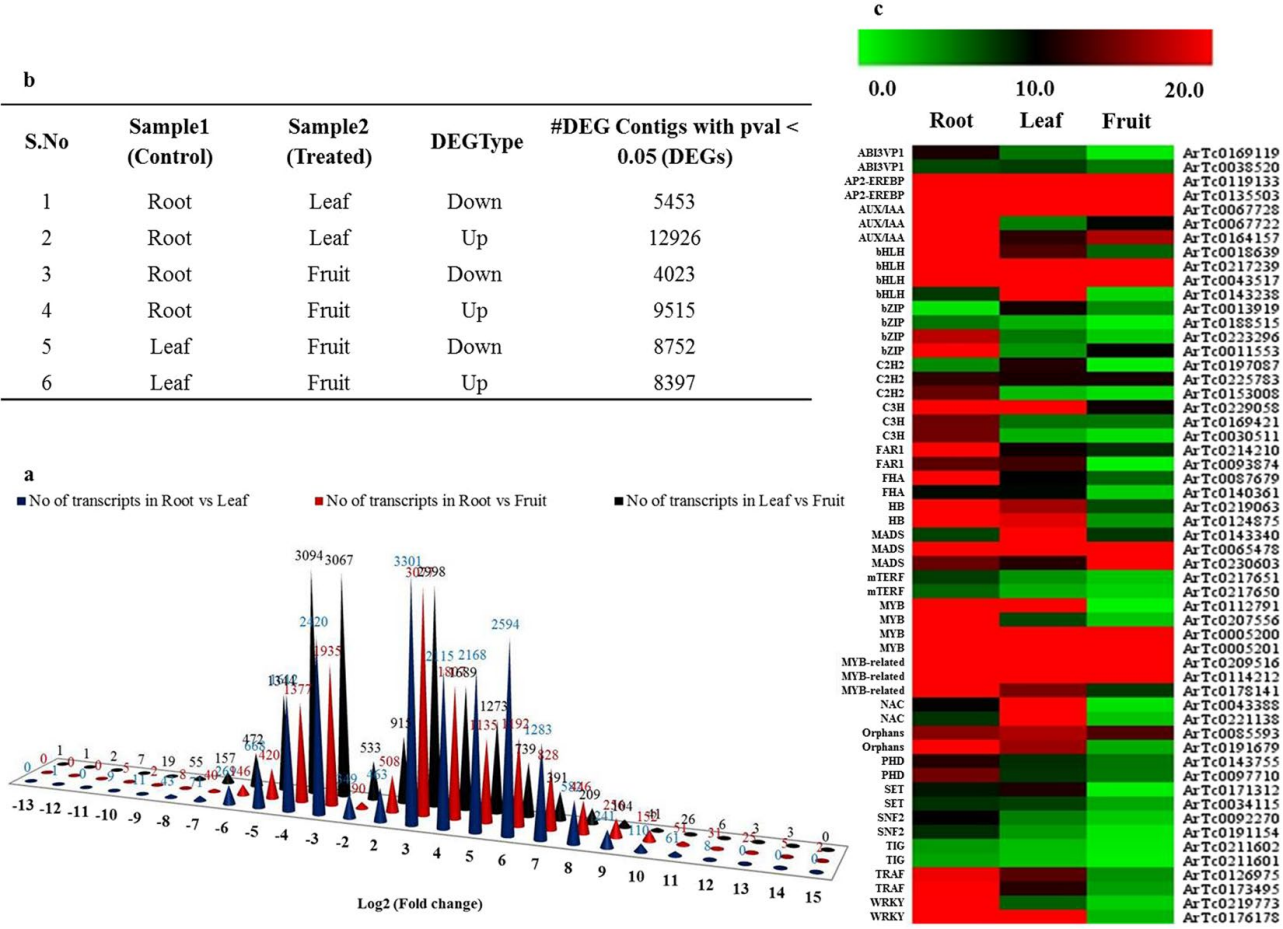
Differential gene expression analysis. (**a)** Pair-wise comparisons of different tissues in all possible combinations to identify differentially expressed transcripts with Log2fold change. (**b**) Total number of transcripts showing differential gene expression between the tissues. (**c)** Differential gene expression analysis of transcription factors involved in secondary metabolite biosynthesis.

RNA-seq has emerged as a very good platform to measure tissue specific gene expression pattern of any organism at the whole-genome level^19,26^. To investigate differential gene expression in individual tissues, all high quality short reads from individual samples were mapped on *A. racemosus* transcriptomes using DEGseq software. A total of 88–95% of high quality short reads were mapped onto *A. racemosus* transcriptome (Table 2). Differentially expressed genes were identified among the different tissues of *A. racemosus* using pairwise alignment with log2fold change (Fig. 6a). A total of 5453 transcripts were down-regulated and 12926 transcripts were up regulated in leaf tissues as compared to the root, whereas 4023 transcripts were down-regulated and 9515 transcripts were up-regulated in fruit tissue. However, nearly equal numbers of transcripts were down-regulated (8752) and up-regulated (8397) in fruit tissue as compared to the leaf (Fig. 6b). Differential expression analysis of the major transcription factors detected and reported to be involved in controlling the secondary metabolite biosynthesis was performed and found to be correlating well with the metabolic profile data (Fig. 6c).

We further performed the GO analysis under biological process, molecular function and cellular component category in which these differentially expressed transcripts are involved. A total of 5253 transcripts were annotated under 37 biological processes, 4481 transcripts under 20 cellular components, and 4647 transcripts under 23 molecular function categories out of 12926 up-regulated transcripts of leaf tissues. However, 1378 transcripts were annotated under 30 biological process categories, 808 transcripts under 21 cellular component categories and 1268 transcripts under 21 molecular function categories in the 5453 down-regulated transcripts in leaf tissues as compared to the root (Figs S18 and 19). In case of the 9515 transcripts up-regulated in fruit as compared to root, 3559 transcripts were annotated under 37 biological process categories, 2518 transcripts under 21 cellular component categories and 2585 transcripts under 23 molecular function categories, whereas, 940 transcripts were annotated under 30 biological process categories, 397 transcripts under 20 cellular component categories and 732 transcripts under 20 molecular function categories in the case of the 4023 down-regulated transcripts in fruit tissues as compared to the root (Figs S20 and 21). Furthermore, in case of the 8752 transcripts down-regulated in fruit as compared to leaf, 3601 transcripts were annotated under 35 biological process categories, 3092 transcripts under 23 cellular component categories and 3280 transcripts under 22 molecular function categories, whereas, 3008 transcripts were annotated under 35 biological process categories, 2207 transcripts under 22 cellular component categories and 2353 transcripts under 23 molecular function categories in the 8397 up-regulated in fruit as compared to leaf (Figs S22 and 23). Top biological categories were represented by 1027 transcripts in biological processes, 923 transcripts in cellular processes, 871 transcripts in metabolic processes and 377 transcripts in biosynthetic processes out of 12926 transcripts up-regulated in leaf as compared to root, whereas top biological categories in down-regulated transcripts (5453) were represented by biological process (293), cellular process (250), metabolic process (233), biosynthetic process (95) and carbohydrate metabolic process (93). However, top five biological categories in up-regulated transcripts in fruit tissues as compared to root were represented by biological process (754), cellular process (622), metabolic process (593), biosynthetic process (270) and carbohydrate metabolic process (192), whereas biological process (187), metabolic process (168), cellular process (164), nucleobase-containing compound metabolic process (102) and DNA metabolic process (75) were the largest biological categories in down-regulated transcripts. Investigation of biological process categories in differentially expressed transcript in leaf as compared to fruit, were represented by cell communication (57), response to stress (56), cellular protein modification process (42), DNA metabolic process (24) and multicellular organism development (22) in up-regulated transcripts, whereas, biological process (699), cellular process (635), metabolic process (583), nucleobase-containing compound metabolic process (264) and biosynthetic process (248) in down-regulated transcripts in leaf as compared to fruits.

### Identification and differential expression analysis of candidate genes involved in the biosynthetic pathway of monoterpenoids, steroidal saponins and triterpene saponins

**Figure 7:**
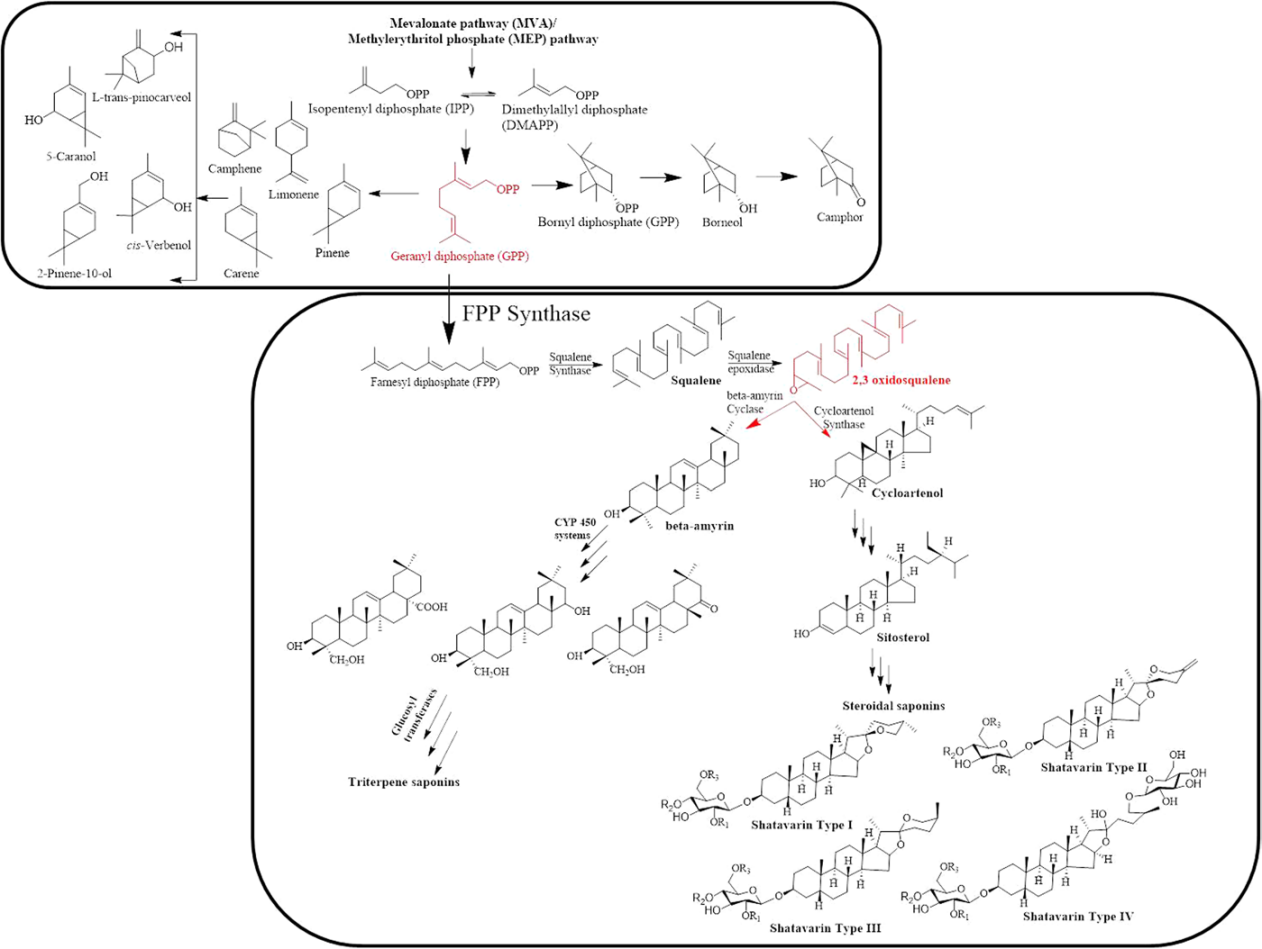
Proposed biosynthetic pathway of terpenoids present in *A. racemosus*.

**Figure 8:**
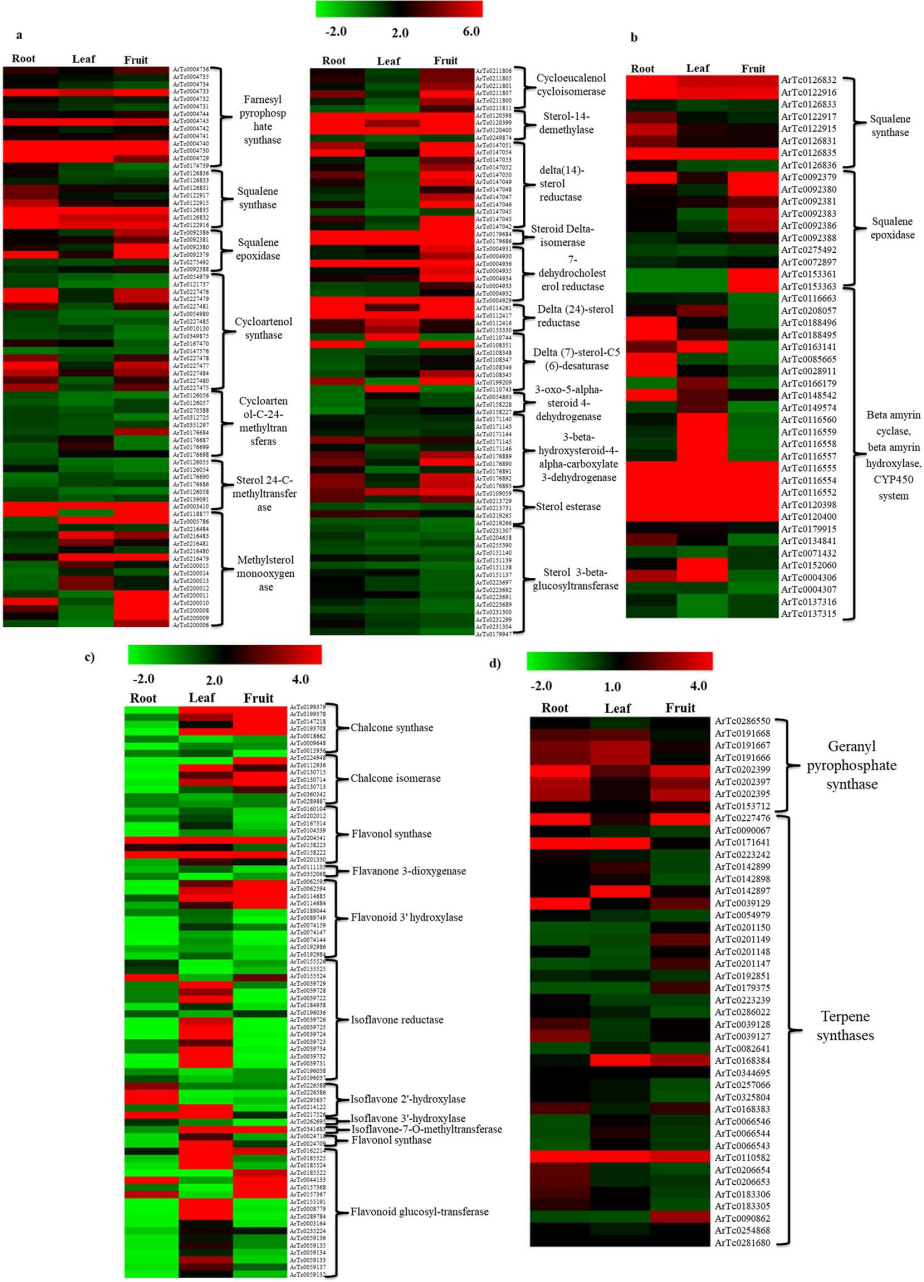
Heatmap of candidate genes involved in the biosynthetic pathway of secondary metabolites detected in various tissues (root, leaf and fruit) of *A. racemosus*. (**a**) Transcripts involved in steroidal saponins biosynthetic pathway. (**b)** Transcripts involved in triterpene saponins biosynthetic pathway. (**c)** Transcripts involved in flavonoid biosynthetic pathway. (**d)** Transcripts involved in monoterpenoid biosynthetic pathway. Color scale showing normalized FPKM values is shown at the top.

As secondary metabolites have immense applications in therapeutics, it is of great interest to establish the biosynthetic pathway of major secondary metabolic content such as steroidal/triterpene saponins, monoterpenoids and flavonoids present in *A. racemosus*. Steroidal saponins are a complex and structurally diverse group of organic compounds consisting of steroidal aglycon backbone (cycloartane triterpenoids) attached with numerous oligosaccharide moieties. They are biosynthesized from two simple five-carbon building blocks, isopentenyl diphosphate (IPP) and dimethylallyl diphosphate (DMAPP), which are produced through either the mevalonate (MVA) pathway or the methylerythritol pathway (MEP)^27–29^. Dimethylallyl diphosphate (DMAPP) condenses with its isomer isopentenyl diphosphate (IPP) in a head to tail fashion to produce higher acyclic prenyl diphosphates such as geranyl diphosphate (GPP), farnesyl diphosphate (FPP), geranylgeranyl diphosphate (GGPP) catalyzed by the prenyl transferases geranyl diphosphate synthase (GDS), farnesyl diphosphate synthase (FDS) and geranylgeranyl diphoshate synthase (GGDS), respectively^30,31^. Farnesyl diphsophate (FPP) acts as the main branching point in isoprenoid biosynthesis of sesquiterpenoids, sterols, brassinosteroids, dolichols and polyprenols^32–34^. The proposed biosynthetic pathway of steroidal saponins starts with the head to head condensation of two units of farnesyl diphosphate to produce a 30 carbon linear squalene catalyzed by squalene synthase. Further squalene is converted into squalene epoxide with the help of squalene epoxidase, which serve as the initial step of triterpene biosynthesis. Squalene epoxide undergoes cyclization with the help of a variety of triterpene cyclases via protonation and epoxide ring opening. The type of cyclases involved in the cyclization of squalene expoxide play a crucial role in deciding the type of molecules it will produce. Squalene epoxide can undergo cyclization with the help of cycloartenol synthase and beta-amyrin synthase to produce tetracyclic cycloartenol and pentacyclic beta-amyrin backbone, which act as precursor for the biosynthesis of steroidal saponin and triterpene saponin, respectively. Furthermore, cycloartenol goes through a series of rearrangements to produce sitosterol, catalyzed by various enzymes such as: cycloeucalenol cycloisomerase, methylsterol monooxygenase, sterol 14 alpha-demethylase, 7-dehydrocholesterol reductases, lanosterol oxidase etc. Sitosterol could act as a direct precursor for the biosynthesis of steroidal saponins and undergoes glucosylation at various positions to produce saponins catalyzed by glucosyltransferases^17,34–36^. However, beta-amyrin gets functionalized with the help of CYP450 systems to generate hydroxyl, epoxy and carboxy derivatives, which are ultimately glucosylated by glucosyltransferases to produce triterpene saponins^36,37^. The proposed biosynthetic pathways of steroidal saponins and triterpene saponins present in *A. racemosus* are depicted in Fig. 7. We have identified the transcripts encoding for all the enzymes catalyzing different intermediate reactions involved in steroidal saponins (153) as well as triterpene saponin biosynthetic pathway (45) in *A. racemosus* transcriptome. Furthermore, we also analyzed the expression pattern of all these transcripts involved in steroidal saponin and triterpene saponin biosynthetic pathways using RNA-Seq data in three different tissue samples. Majority of the genes of steroidal saponin biosynthetic pathway were found to be up-regulated in root as compared to other tissues. However, majority of the transcripts belonging to triterpene saponin biosynthetic pathway were found to be up-regulated in fruit and leaf tissues as compared to root (Fig. 8a,b). We have also identified 129 unique transcripts belonging to terpenoid backbone biosynthetic pathway (MVA/MEP) and their differential expression pattern was established (Fig. S24). The expression pattern of the genes is very much in correlation with the metabolic profile of the steroidal and triterpene saponins in various tissues of *A. racemosus*. This could be a result of co-ordinated regulation of all these pathways derived from a common precursor. Similar expression patterns have been reported earlier for steroidal saponin biosynthetic pathway genes from *A. racemosus* in leaf and root tissues^17^. Nevertheless, we report here a more comprehensive analysis of steroidal saponin biosynthesis along with triterpene saponin biosynthesis, which is originated from the same precursor in three different tissues.

The LC-MS analysis resulted in the identification of a large number of flavonoids differentially present in the *A. racemosus*. We screened *A. racemosus* transcriptome for all the genes involved in the biosynthesis of flavonoids and its glycosides. The biosynthetic pathway of flavonoids start with the condensation of one molecule of 4-coumaroyl-CoA and three molecules of malonyl CoA producing naringenin chalcone catalyzed by chalcone synthase (CHS). The naringenin chalcone is subsequently converted into naringenin with the help of chalcone isomerase (CHI). The oxidation of the naringenin catalyzed by flavanone 3-hydroxylase (F3H) leads to the formation of dihydrokaempferol which can be hydroxylated on the 3′ position of the B-ring, by flavonoid 3′-hydroxylase (F3′H) producing dihydroquercetin. Dihydrokaempferol and dihydroquercetin subsequently get converted into kaempferol and quercetin, respectively, by flavonol synthase^38,39^. Quercetin undergoes glucosylation with the help of glucosyltransferases to produce quercetin glycosides (rutin and hyperoside). Numerous efforts have been made towards understanding and establishing the biosynthesis of flavonoids in a variety of plant species with the genetic perspective, especially in *Arabidopsis thaliana* and *Zea mays*^40,41^. Although the basic biosynthetic pathway of flavonoids is highly conserved in plants, groups of enzymes belonging to isomerases, reductases and hydroxylases modify the basic skeleton leading to the formation of diverse class of flavonoids^42^. We have identified 61 unique transcripts involved in the biosynthesis of aglycon portion of flavonoids and 18 transcripts belonging to glucosyltransferases, which might be involved in the biosynthesis of glucosylated flavonoids and established their expression patterns (Fig. 8c).

Metabolic analysis of the n-hexane fraction of all three tissues by GC-MS resulted in the identification of a large number of compounds belonging to monoterpenoids exclusively in the root tissues. Monoterpenoids are biosynthesized by conversion of geranyl diphosphate into the monoterpene backbone catalyzed by monoterpene synthases. These monoterpene backbones can undergo functionalization to produce active monoterpenoids with the help of CYP450 systems^43–45^. In order to establish the biosynthesis of these important compounds in *A. racemosus* root, we have identified the candidate genes responsible for monoterpenoid biosynthesis in *A. racemosus* transcriptome. A total of 36 transcripts belonging to the terpene synthase family and 8 transcripts belonging to geranyl transferases (GPP synthase) were identified based upon functional annotation of *A. racemosus* transcriptome against various publically available databases. The terpene synthases identified were found to match with limonene synthase, terpineol synthase, ocimene synthase, borneol dehydrogenase, etc. These terpene synthases might be responsible for the accumulation of terpenoids/monoterpenoids in *A. racemosus* root. Differential gene expression analysis of genes encoding terpene synthases revealed the presence of these terpene synthases in all the three tissues with substantial difference in the expression pattern (Fig. 8d). Although these terpene synthases are present in all the tissues, monoterpenoids were detected in only root. These findings suggest that, there could be some other regulatory mechanisms, which might be responsible for the biosynthesis/accumulation of monoterpenoids in only the root tissues.

### Real-time PCR for validation of differentially expressed genes

Real-time PCR was performed to validate 8 selected genes from RNA-seq data involved in the biosynthesis of monoterpeneoids, steroidal saponins, triterpene saponin, flavonoids and transcription factors, which are as follows: trans-ocimene synthase (ArTc0039129), geraniol 8-hydroxylase (ArTc0168384), cycloartenol synthase (ArTc0227479), beta-amyrin hydroxylase (ArTc0116555), chalcone synthase (ArTc0199379), sterol glucosyltransferase (ArTc0231304), flavonoid glucosyltransferase (ArTc0162214) and WRKY transcription factor (ArTc0010047) in two biological replicates for all the three samples (Fig. S25). Comparative expression level analysis of all selected genes revealed similar expression pattern as observed from RNA-seq data analysis.

## Conclusion

The metabolic fingerprinting of three different tissues of *A. racemosus* using GC-MS and LC-MS have led to the identification of a large number of monoterpenoids, steroidal/triterpene saponins and flavonoids which have not been reported earlier. Our findings clearly indicated that where monoterpenoids are exclusively present in the root, steroidal saponins are dominated in leaf and root tissues, whereas triterpene saponins and flavonoids are dominated in leaf and fruit tissues. Deep transcriptome sequencing and comprehensive analysis resulted in the identification of all the transcripts encoding enzymes involved in biosynthesis/accumulation of steroidal/ triterpene saponins, monoterpenoids and flavonoids in *A. racemosus*. Differential expression analysis of candidate genes revealed that expression of most of the transcripts involved in steroidal saponin biosynthesis is restricted to leaf and root tissues, whereas triterpene saponins and flavonoids biosynthesis is dominated in leaf and fruit tissues. However, the transcripts involved in monoterpenoids biosynthesis are present in all the tissues. The expression profiles of these pathway genes are well in agreement with the metabolic content of the plant. Elucidation of biosynthetic pathway of active components and establishing the expression profiling of candidate genes that encode for enzymes and transcription factors will pave the way for revealing the regulatory mechanism for their biosynthesis in *A. racemosus*.

## Materials and Methods

### Plant material and metabolite extraction

Various parts (roots, leaves and fruits) of *A. racemosus* were collected from the wild near Mulshi Dam, Pune, India, and stored at −80 °C till further use. A schematic representation of the procedure for the metabolite extraction with slight modification^46^ and analysis using GC-MS and LC-MS for the metabolite characterization is presented in supplementary material (Fig. S26). The non-polar (n-hexane) and polar (75% methanol) extracts from all the three tissues (root, leaf and fruit) were injected in GC-MS and LC-MS for the metabolite analysis.

### GC-MS/LC-MS analysis

1 µL of n-hexane extracts of all the tissue samples (root, leaf and fruit) were injected in GC-MS equipped with a 30 m × 0.32 mm × 0.30 µm capillary column (HP-5) and a FID. The column was equilibrated at 50 °C followed by a temperature gradient from 50 to 120 °C at 2 °C/min, followed by a second temperature gradient of 10 °C/min from 120 to 180 °C and a final hold at 180 °C for 5 min with helium as the carrier gas at a flow rate of 1 mL/min. All the products formed were characterized by comparing the mass fragmentation pattern with NIST/Wiley mass spectral library. LC-MS analysis was performed using Thermo Q-Exactive benchtop HESI-HRMS equipped with 2.6 µ silica column (C18) (Thermo accucore) of 15 cm length in positive mode. Gradient elution was performed starting with 5% ACN (Solvent A) and 0.1% formic acid buffered H_2_O (Solvent B) to 100% ACN over 25 minutes and column was re-equilibrated at 5% ACN for 5 minutes at a flow rate of 0.5 mL/minute. Non-targeted data analysis was performed using R based XCMS software for peak alignment, retention time correction and peak picking. Adduct annotation for mass features were performed with CAMERA and final mass features were mapped to KEGG data base using ProbMetab software^47,48^ to assign the putative identification of metabolites present in *A. racemosus*.

### RNA isolation and transcriptome sequencing

RNA was isolated from the root, leaf and fruit tissues of *A. racemosus* using Spectrum™ Plant Total RNA Kit (Sigma-Aldrich) according to the manufacturer’s instructions from two biological replicates. The quantity and quality of isolated total RNA was determined by Nanodrop and Bioanalyzer. The high quality total RNA from all the three tissues (root, leaf and fruit) was sent for RNA sequencing to Sci-Genom Labs, Cochin, India. High quality (A_260/280_, 1.8–2.0, A_260/230_ > 2.0 and RIN > 6.5) total RNA from each sample was used for transcriptome sequencing using Illumina GAII analyzer platform at Sci-Genom Labs, Cochin, India. To obtain high-quality clean read data for *de novo* transcriptome assembly, stringent primary QC check was performed to remove low quality read and adapter trimming of the raw data using the in-built NGS QC Toolkit-v2.3.

### *De novo* transcriptome assembly

High-quality reads were assembled into contigs using various commonly used short-read assemblers, such as Trinity (v2012-05-18)^49^, Velvet-Oases (v0.2.04)^50^, and SOAPdenovo (v1.04) with default parameters. The assembly obtained from all the three assemblers were subjected to CD-HIT tool for the removal of redundancy. The non-redundant transcripts were used for the functional annotation and expression profiling.

### Functional annotation

To assign the putative function to each transcript of *A. racemosus*, similarity search was performed using BLASTX search^51^ against SwissProt/Uniprot and NCBI non-redundant databases with an E-value cut-off of ≤10^−5^ to find the best significant match for each transcript. Based on the above sequence comparisons, GO terms were assigned to each *A. racemosus* transcripts under molecular function, biological process and cellular component categories. To identify the specific biosynthetic pathways present in *A. racemosus*, all the transcripts were submitted to KEGG (Kyoto Encyclopedia of Genes and Genomes) database to assign KO (KEGG Orthology) number and generate KEGG pathways with default parameters^52^. Identification of TF families in *A. racemosus* transcriptome was carried out based on Hidden Markov Model (HMM) profile search. *A. racemosus* transcriptome was also scanned for the presence of simple sequences repeats (SSR) using MISA (MIcroSAtellite), a Perl script program with default parameters^53^. The number of repeating units in the present study for mono and di-nucleotide was considered to be six, whereas for tri-, tetra-, penta- and hexa-nucleotide repeats was more than five in the search criteria.

### Differential gene expression analysis

To estimate the expression pattern for each transcript in individual tissue sample, high-quality reads from all the samples were mapped on the transcriptome assembly using Bowtie2 program. A maximum of 1-mismatches was allowed in the seed region (length = 31 bp) and all multiple mapped positions were reported. The unique read counts for each tissue was normalized by calculating fragment per kilobase per million (FPKM) for each transcript. Differential gene expression analysis was performed using DESeq program (v1.10.1)^54^. A *P*-value cut-off of ≤0.05 along with at least two-fold change was used as criteria to identify differential gene expression pattern. All the heatmaps showing tissue-specific expression patterns for the transcripts involved in various pathways were generated via MultiExperiment Viewer (MeV, v4.8).

### Real-time PCR analysis

For real-time PCR analysis, all the gene specific primers were designed using Primer Express (V3.0) software listed in table S4. Real-time PCR was carried out in three independent technical replicates for each tissue sample for two biological replicates in a total reaction volume of 25 µL using 20 ng of cDNA for each selected gene. GAPDH was used as an internal control in each sample for normalization.

### Data availability

The raw sequence data of *Asparagus racemosus* transcriptome from all the three tissues (root, leaf and fruit) have been deposited to the SRA NCBI database with the accession numbers SRR6161788, SRR6161787 and SRR6161789, respectively.

## Electronic supplementary material

Supplementary information
