# Supplementary material for: Comprehensive metabolic and transcriptomic profiling of various tissues provide insights for saponin biosynthesis in the medicinally important Asparagus racemosus
Source: Sci Rep. 2018 Jun 14;8:9098. doi: 10.1038/s41598-018-27440-y (PMC6002474; doi:10.1038/s41598-018-27440-y)
Supplement: Supplementary file 1 — Supplementary information [file 41598_2018_27440_MOESM1_ESM.docx]

**Supplementary Information**

**Comprehensive metabolic and transcriptomic profiling of various tissues provide insights for saponin biosynthesis in the medicinally important *Asparagus racemosus***

Prabhakar Lal Srivastava^1^*, Anurag Shukla^2^, Raviraj M. Kalunke^2^

^1^Symbiosis School of Biological Sciences, Symbiosis International (Deemed University), Lavale, Pune-412115, India.

^2^Biochemical Sciences Division, CSIR-National Chemical Laboratory, Dr. Homi Bhabha Road, Pashan, Pune-411008, India.

*Correspondence should be addressed to: [prabhakarbiotech@gmail.com](mailto:prabhakarbiotech@gmail.com),

**Table S1.** Saponins and flavonoids detected in LCMS profile of various extracts of *Asparagus racemosus*

| **Peak No.** | **Exact Mass** | **Observed Mass** | **Observed mass after adduct correction** | **Mass error in PPM** | **Retention time (min)** | **KEGG ID** |
| --- | --- | --- | --- | --- | --- | --- |
| **Steroidal saponins** | | | | | | |
| 1 | 740.4331 | 741.4404 | 740.4326 | -0.7496 | 6.85 | C17075 |
| 2 | 740.4287 | 741.4360 | 740.4282 | -0.7358 | 7.08 | C17075 |
| 3 | 740.4337 | 741.4410 | 740.4332 | -0.7568 | 9.18 | C17075 |
| 4 | 740.4338 | 741.4411 | 740.4333 | -0.7229 | 11.54 | C17075 |
| 5 | 740.4337 | 741.4410 | 740.4332 | -0.7375 | 12.16 | C17075 |
| 6 | 740.4315 | 741.4388 | 740.4310 | -0.7383 | 16.22 | C17075 |
| 7 | 884.4748 | 885.4821 | 884.4743 | -0.5734 | 6.97 | C08901 |
| 8 | 884.4755 | 885.4828 | 884.4750 | -0.5822 | 7.20 | C08901 |
| 9 | 884.4762 | 907.4650 | 884.4753 | -1.5558 | 9.65 | C08894 |
| 10 | 884.4758 | 885.4830 | 884.4752 | -0.6472 | 9.82 | C08894 |
| 11 | 884.4761 | 907.4651 | 884.4754 | -1.4649 | 10.23 | C08894 |
| 12 | 884.4771 | 867.4724 | 884.4751 | -2.2733 | 10.61 | C08901 |
| 13 | 884.4761 | 885.4834 | 884.4756 | -0.6284 | 11.15 | C08894 |
| 14 | 884.4754 | 907.4650 | 884.4753 | -0.0636 | 12.24 | C08894 |
| 15 | 884.4737 | 885.4809 | 884.4731 | -0.6500 | 17.38 | C08894 |
| 16 | 886.4841 | 887.4905 | 886.4827 | -1.6102 | 7.06 | C08912 |
| 17 | 886.4882 | 887.4955 | 886.4877 | -0.5988 | 7.24 | C08905 |
| 18 | 886.4917 | 887.4990 | 886.4912 | -0.6267 | 11.59 | C08912 |
| 19 | 902.4860 | 903.4932 | 902.4854 | -0.6534 | 6.76 | C17470 |
| 20 | 902.4860 | 903.4933 | 902.4855 | -0.5775 | 7.84 | C17470 |
| 21 | 902.4846 | 925.4755 | 902.4858 | 1.3430 | 9.73 | C17470 |
| 22 | 902.4828 | 903.4901 | 902.4823 | -0.6274 | 15.41 | C17470 |
| 23 | 932.4679 | 891.4723 | 932.4750 | 7.6150 | 13.14 | C17921 |
| 24 | 944.5290 | 473.2718 | 944.5279 | -1.0063 | 15.63 | C08792 |
| 25 | 1048.5442 | 1049.5520 | 1048.5442 | -0.0450 | 6.63 | C08907 |
| 26 | 1048.5416 | 525.2781 | 1048.5405 | -0.9539 | 6.97 | C08907 |
| 27 | 1062.5243 | 1063.5320 | 1062.5242 | -0.0816 | 6.64 | C08887 |
| 28 | 1062.5230 | 1063.5300 | 1062.5222 | -0.7500 | 7.70 | C08887 |
| 29 | 1062.5238 | 1063.5310 | 1062.5232 | -0.8842 | 9.77 | C08887 |
| 30 | 1062.5224 | 1063.5300 | 1062.5222 | -0.2117 | 13.61 | C08887 |
| 31 | 1064.5397 | 1065.5470 | 1064.5392 | -0.4687 | 5.93 | C08895 |
| 32 | 1064.5408 | 1047.5360 | 1064.5387 | -2.0268 | 6.21 | C08908 |
| 33 | 1066.5742 | 1067.5810 | 1066.5732 | -0.9117 | 5.75 | C15698 |
| **Triterpene Saponins** | | | | | | |
| 1 | 1030.5340 | 1031.5410 | 1030.5332 | -0.7713 | 8.27 | C17469 |
| 2 | 1030.5341 | 1053.5230 | 1030.5333 | -1.0235 | 11.76 | C17469 |
| 3 | 1030.5341 | 1031.5410 | 1030.5332 | -0.9130 | 12.24 | C17469 |
| 4 | 1030.5337 | 1031.5410 | 1030.5332 | -0.4576 | 7.54 | C08960 |
| 5 | 1030.5338 | 1031.5410 | 1030.5332 | -0.5768 | 7.05 | C17469 |
| 6 | 1104.5344 | 1105.5420 | 1104.5342 | -0.1871 | 8.02 | C08934 |
| 7 | 1104.5343 | 1105.5420 | 1104.5342 | -0.1482 | 5.45 | C08934 |
| 8 | 1104.5334 | 1105.5410 | 1104.5332 | -0.2058 | 6.51 | C08934 |
| 9 | 1104.5343 | 1087.5300 | 1104.5327 | -1.4751 | 7.20 | C08934 |
| 10 | 1130.5458 | 1131.5530 | 1130.5452 | -0.5161 | 8.09 | C08921 |
| 11 | 1130.5488 | 1131.5570 | 1130.5492 | 0.3504 | 7.74 | C08921 |
| 12 | 1146.5452 | 1147.5520 | 1146.5442 | -0.8652 | 7.57 | C08933 |
| 13 | 926.4869 | 927.4941 | 926.4863 | -0.6402 | 13.37 | C17540 |
| 14 | 926.4867 | 949.4754 | 926.4857 | -1.1477 | 13.13 | C17545 |
| 15 | 868.4810 | 869.4883 | 868.4805 | -0.5763 | 12.49 | C08940 |
| 16 | 868.4805 | 869.4878 | 868.4800 | -0.6412 | 10.81 | C08897 |
| **Flavonoids** | | | | | | |
| 1 | 290.0763 | 291.0836 | 290.0758 | -1.9175 | 1.30 | C05907 |
| 2 | 743.1970 | 744.2041 | 743.1963 | -0.7347 | 1.42 | C08612 |
| 3 | 534.0993 | 535.1066 | 534.0988 | -0.9366 | 2.59 | C10103 |
| 4 | 918.2449 | 307.0889 | 918.2432 | -1.8395 | 3.48 | C12636 |
| 5 | 772.2053 | 773.2126 | 772.2048 | -0.6479 | 3.76 | C12635 |
| 6 | 919.2473 | 307.4230 | 919.2455 | -1.9301 | 4.16 | C16294 |
| 7 | 372.1201 | 373.1274 | 372.1196 | -1.5088 | 4.20 | C10186 |
| 8 | 578.1607 | 579.1680 | 578.1602 | -0.8728 | 4.22 | C12628 |
| 9 | 316.0589 | 317.0661 | 316.0583 | -1.8474 | 4.46 | C04443 |
| 10 | 772.2052 | 773.2126 | 772.2048 | -0.6889 | 5.02 | C12635 |
| 11 | 304.0555 | 305.0628 | 304.0550 | -1.8562 | 5.10 | C05911 |
| 12 | 610.1523 | 611.1600 | 610.1522 | -0.8887 | 5.26 | C12634 |
| 13 | 548.1887 | 549.1960 | 548.1882 | -0.9742 | 5.46 | C15579 |
| 14 | 464.0952 | 465.1025 | 464.0947 | -1.1775 | 5.50 | C05623 |
| 15 | 302.0421 | 303.0494 | 302.0416 | -1.7201 | 5.51 | C00389 |
| 16 | 372.1205 | 373.1278 | 372.1200 | -1.3789 | 5.53 | C10190 |
| 17 | 594.1582 | 595.1655 | 594.1577 | -0.8830 | 5.55 | C12630 |
| 18 | 448.1000 | 449.1073 | 448.0995 | -1.1719 | 5.55 | C01821 |
| 19 | 286.0472 | 287.0545 | 286.0467 | -1.9893 | 5.55 | C05903 |
| 20 | 594.1579 | 595.1652 | 594.1574 | -0.8711 | 5.71 | C12630 |
| 21 | 286.0473 | 287.0546 | 286.0468 | -1.7901 | 5.81 | C05903 |
| 22 | 448.1000 | 449.1073 | 448.0995 | -1.1674 | 5.97 | C01821 |
| 23 | 462.1157 | 463.1230 | 462.1152 | -1.1490 | 6.23 | C05990 |
| 24 | 402.1309 | 403.1382 | 402.1304 | -1.3519 | 6.26 | C04007 |
| 25 | 284.0680 | 285.0753 | 284.0675 | -2.0049 | 6.26 | C00814 |
| 26 | 340.1304 | 341.1377 | 340.1299 | -1.6246 | 6.93 | C18023 |
| 27 | 464.1287 | 465.1360 | 464.1282 | -1.0721 | 7.14 | C16422 |
| 28 | 432.1048 | 455.0941 | 432.1044 | -1.0357 | 7.24 | C01460 |
| 29 | 356.1253 | 357.1326 | 356.1248 | -1.4762 | 7.95 | C18024 |
| 30 | 272.0680 | 273.0753 | 272.0675 | -1.8849 | 8.39 | C00509 |
| 31 | 286.0837 | 287.0910 | 286.0832 | -1.8331 | 9.66 | C02922 |

**Table S2:** Transcriptome assembly stats

| **Assembly** | **Assemblies stats** | | | **Assemblies stats after CD-HIT-Test** | | |
| --- | --- | --- | --- | --- | --- | --- |
|  | **Trinity** | **Velvet-Oasis** | **SOAPdenovoTrans** | **Trinity** | **Velvet-Oasis** | **SOAPdenovoTrans** |
| **Number of contigs** | 459981 | 277091 | 360695 | 362213 | 206004 | 349806 |
| **Number of contigs in scaffolds** | 0 | 6833 | 233531 | 0 | 4116 | 222832 |
| **Number of contigs not in scaffolds** | 459981 | 270258 | 127164 | 362213 | 201888 | 126974 |
| **Total size of contigs** | 3.9E+08 | 361623955 | 178061967 | 282128834 | 249579685 | 170564995 |
| **Longest contig** | 22819 | 44958 | 16888 | 22819 | 44958 | 16624 |
| **Shortest contig** | 201 | 1 | 100 | 201 | 1 | 100 |
| **Number of contigs > 1K nt** | 107920 | 110787 | 38245 | 74878 | 74221 | 36012 |
| **Percentage of contigs > 1K nt** | 23.5 | 40 | 10.6 | 20.7 | 36 | 10.3 |
| **Number of contigs > 10K nt** | 335 | 643 | 18 | 222 | 453 | 13 |
| **Percentage of contigs > 10K nt** | 0.1 | 0.2 | 0 | 0.1 | 0.2 | 0 |
| **Number of contigs > 100K nt** | 0 | 0 | 0 | 0 | 0 | 0 |
| **Percentage of contigs > 100K nt** | 0 | 0 | 0 | 0 | 0 | 0 |
| **Number of contigs > 1M nt** | 0 | 0 | 0 | 0 | 0 | 0 |
| **Percentage of contigs > 1M nt** | 0 | 0 | 0 | 0 | 0 | 0 |
| **Number of contigs > 10M nt** | 0 | 0 | 0 | 0 | 0 | 0 |
| **Percentage of contigs > 10M nt** | 0 | 0 | 0 | 0 | 0 | 0 |
| **Mean contig size** | 851 | 1305 | 494 | 779 | 1212 | 488 |
| **Median contig size** | 397 | 700 | 314 | 380 | 603 | 313 |
| **N50 contig length** | 1655 | 2444 | 702 | 1420 | 2351 | 685 |
| **L50 contig count** | 63228 | 43649 | 67688 | 50663 | 30800 | 66721 |
| **contig %A** | 30.32 | 30.74 | 30.42 | 30.23 | 30.75 | 30.43 |
| **contig %C** | 19.76 | 19.97 | 19.52 | 19.79 | 19.99 | 19.52 |
| **contig %G** | 19.91 | 19.33 | 19.43 | 20.02 | 19.34 | 19.43 |
| **contig %T** | 30 | 29.89 | 30.28 | 29.96 | 29.85 | 30.28 |
| **contig %N** | 0 | 0.07 | 0.35 | 0 | 0.06 | 0.34 |
| **contig %non-ACGTN** | 0 | 0 | 0 | 0 | 0 | 0 |
| **Number of contig non-ACGTN nt** | 0 | 0 | 0 | 0 | 0 | 0 |

**Table S3:** Summary of mapped reads on to the assembly generated using all the three assemblers

| **Samples** | **Alignment % with Velvet-Oasis assembly after CD_HIT** | **Alignment % with Trinity Assembly after CD_HIT** | **Alignment % with Soapdenovo-trans after CD_HIT** |
| --- | --- | --- | --- |
| Root-B1 | 73.19 % | 94.28 % | 65.52 % |
| Root-B2 | 71.81 % | 95.01 % | 64.11 % |
| Leaf-B1 | 70.91 % | 88.27 % | 64.00 % |
| Leaf-B2 | 76.07 % | 91.54 % | 67.44 % |
| Fruit-B1 | 45.27 % | 90.16 % | 40.07 % |
| Fruit-B2 | 27.48 % | 91.62 % | 25.62 % |

**Table S4:** List of primers used for Real-time PCR analysis.

|  |  | Forward (5'-3') | Reverse (5'-3') |
| --- | --- | --- | --- |
| ArTc0039129 |  | GGAGACCCTCCCAGACTACTT | TGAAGTCTGTGGCCTCCCAT |
| ArTc0168384 |  | TGAACTCGGAAATGCGACTGA | ACAAAGGTGGATGCCCAGAG |
| ArTc0227479 |  | ATTGCAGAGATCGAAAGGGCT | CTTGCTCCGATAGCCTGATGT |
| ArTc0116555 |  | GGGTGTGGTCTTGGTCTCAA | CCAACCTCCTCATCTCAACAGT |
| ArTc0199379 |  | TACCCACCTTGACAGCATGG | CCCCTCAGAGTCGGGAAGTA |
| ArTc0010047 |  | TGAAGCTAGCCTCTCCCTCC | GGTTCGGATCCTCTACCTGC |
| ArTc0231304 |  | GGGTGACTGGAGCGGTAAAA | GTCCCCTATTATGGGTGGTTGT |
| ArTc0162214 |  | GAGCTCTTCAAGCGGGTGAT | GGACGTAGTCAGGCTCCAAC |
| ArTc0050733  (GAPDH) |  | ATCTGCAGCTCCTCTTGTCG | ATACCTTGCAGGATTCGGGG |

**
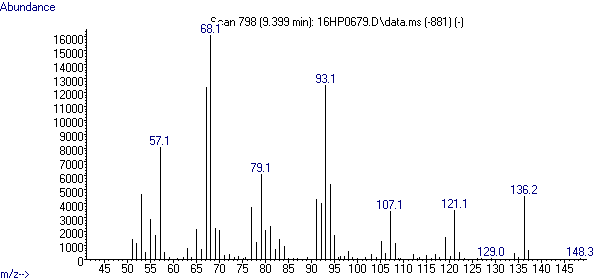
**

**Figure S1:** EI Mass spectrum Limonene (**7**).

**
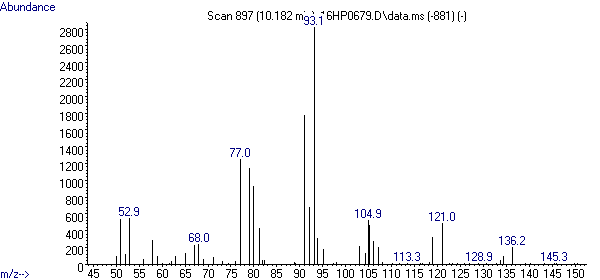
**

**Figure S2:** EI Mass spectrum alpha-Pinene (**13**).


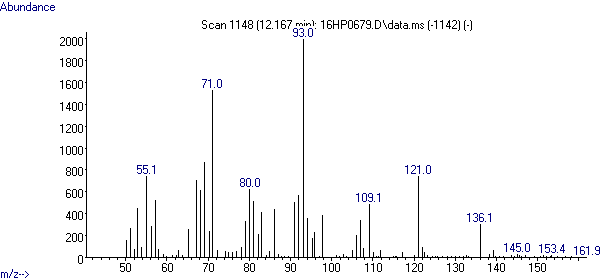


**Figure S3:** EI Mass spectrum Ocimene (**10**).


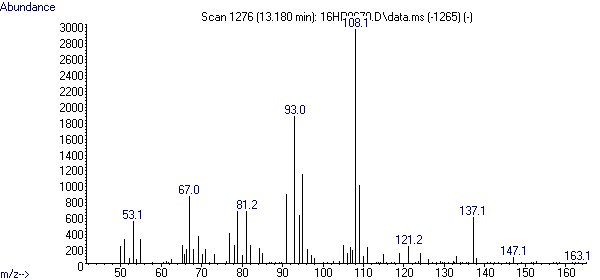


**Figure S4:** EI Mass spectrum Camphene-6-ol (**11**).


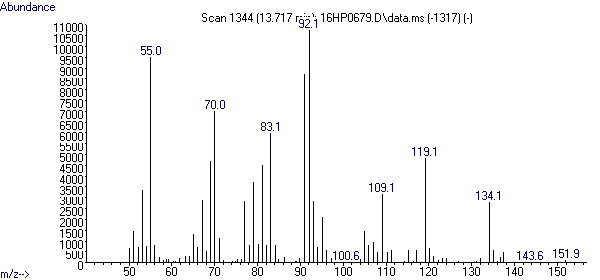


**Figure S5:** EI Mass spectrum L-*trans*-Pinocarveol (**6**).


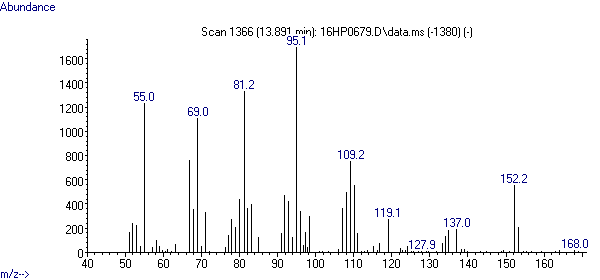


**Figure S6:** EI Mass spectrum (-)-Camphor (**12**).


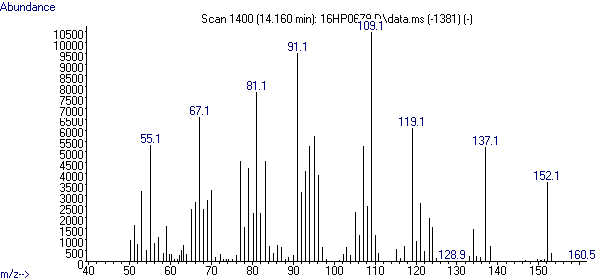


**Figure S7:** EI Mass spectrum d-Verbenol (**5**).


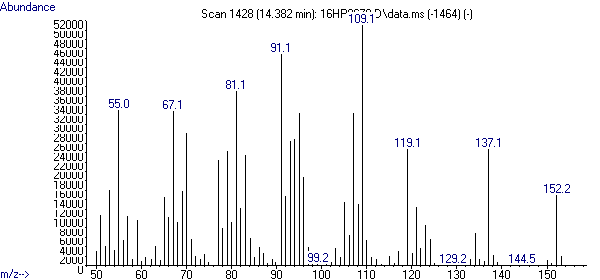


**Figure S8:** EI Mass spectrum (S)-*cis*-Verbenol (**2**).


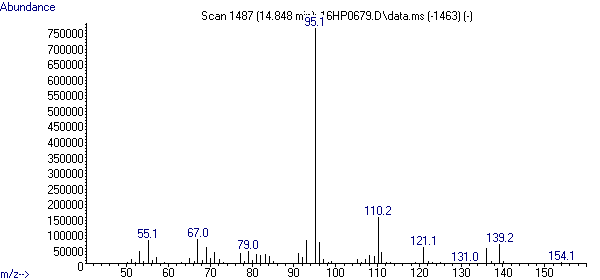


**Figure S9:** EI Mass spectrum (-)-Borneol (**1**).


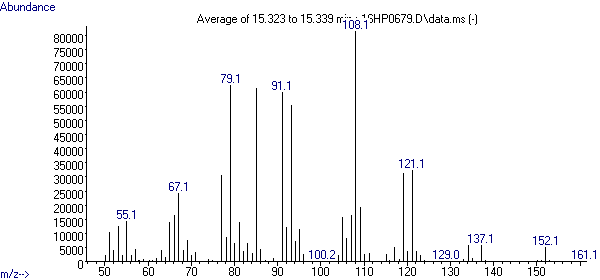


**Figure S10:** EI Mass spectrum 2-Pinen-10-ol (**3**).


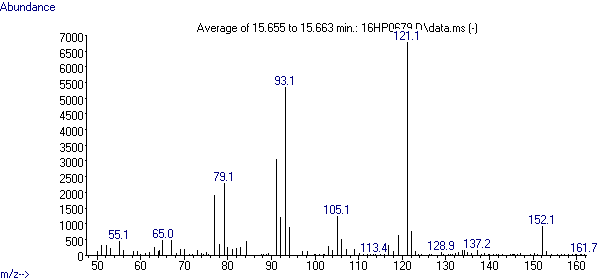


**Figure S11:** EI Mass spectrum Teresantalol (**8**).

**
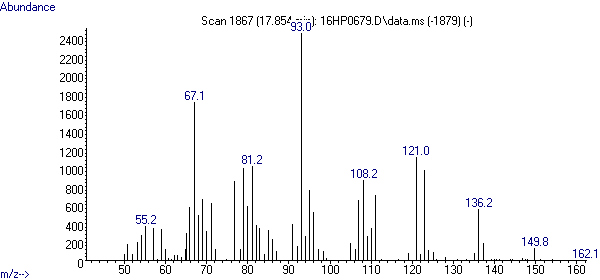
**

**Figure S12:** EI Mass spectrum *cis*-Myrtanol (**9**).

**
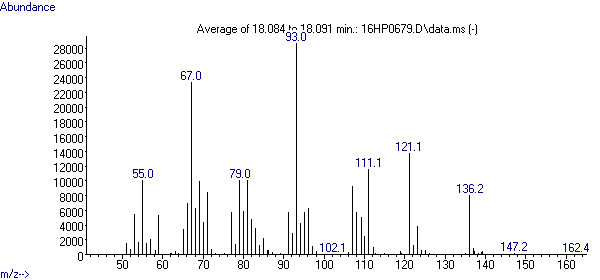
**

**Figure S13:** EI Mass spectrum of 5-Caranol (**4**)


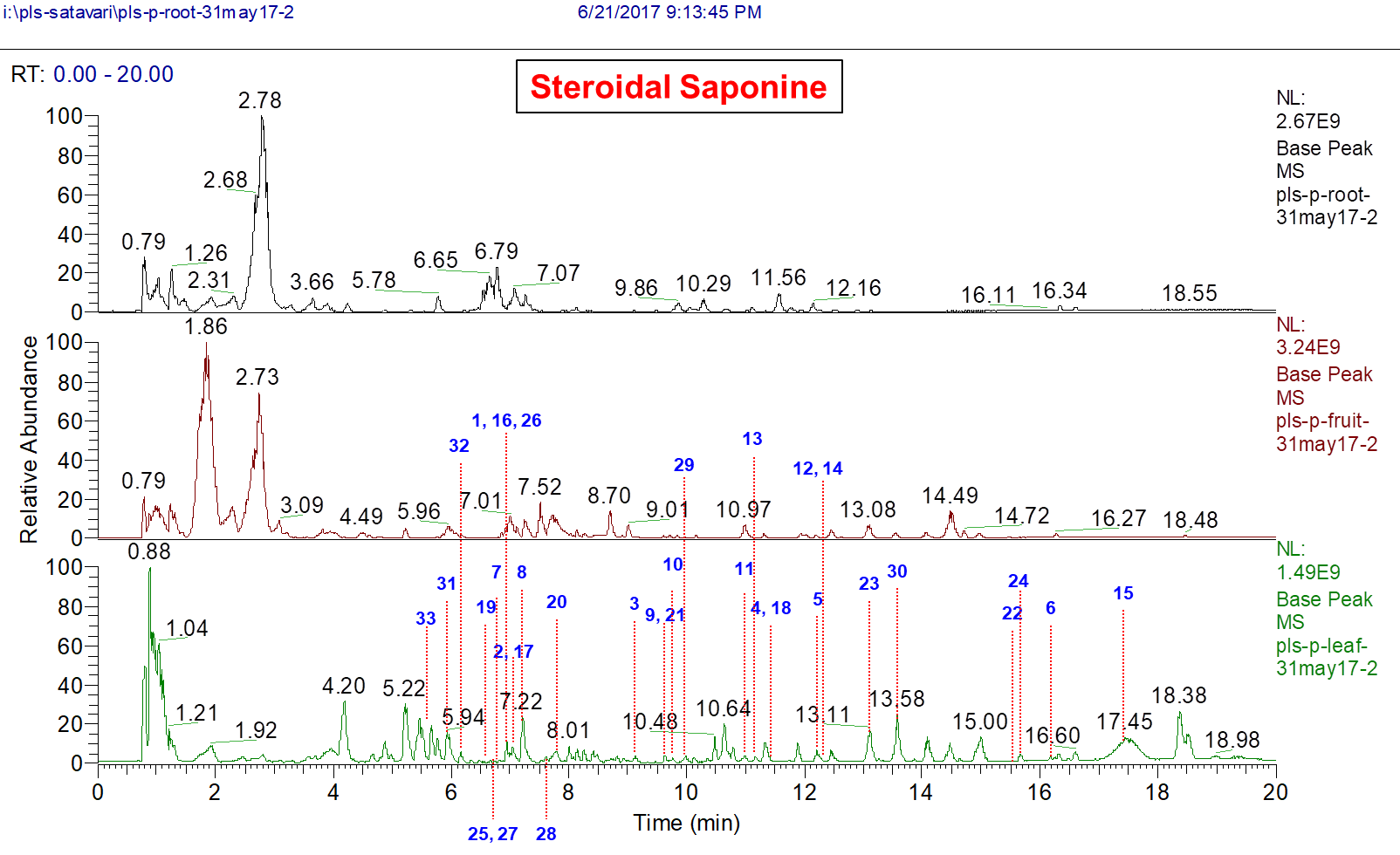


**Figure S14A:** LCMS profiles of extracts of root, leaf and fruit tissues of *A. racemosus* highlighting steroidal saponins.


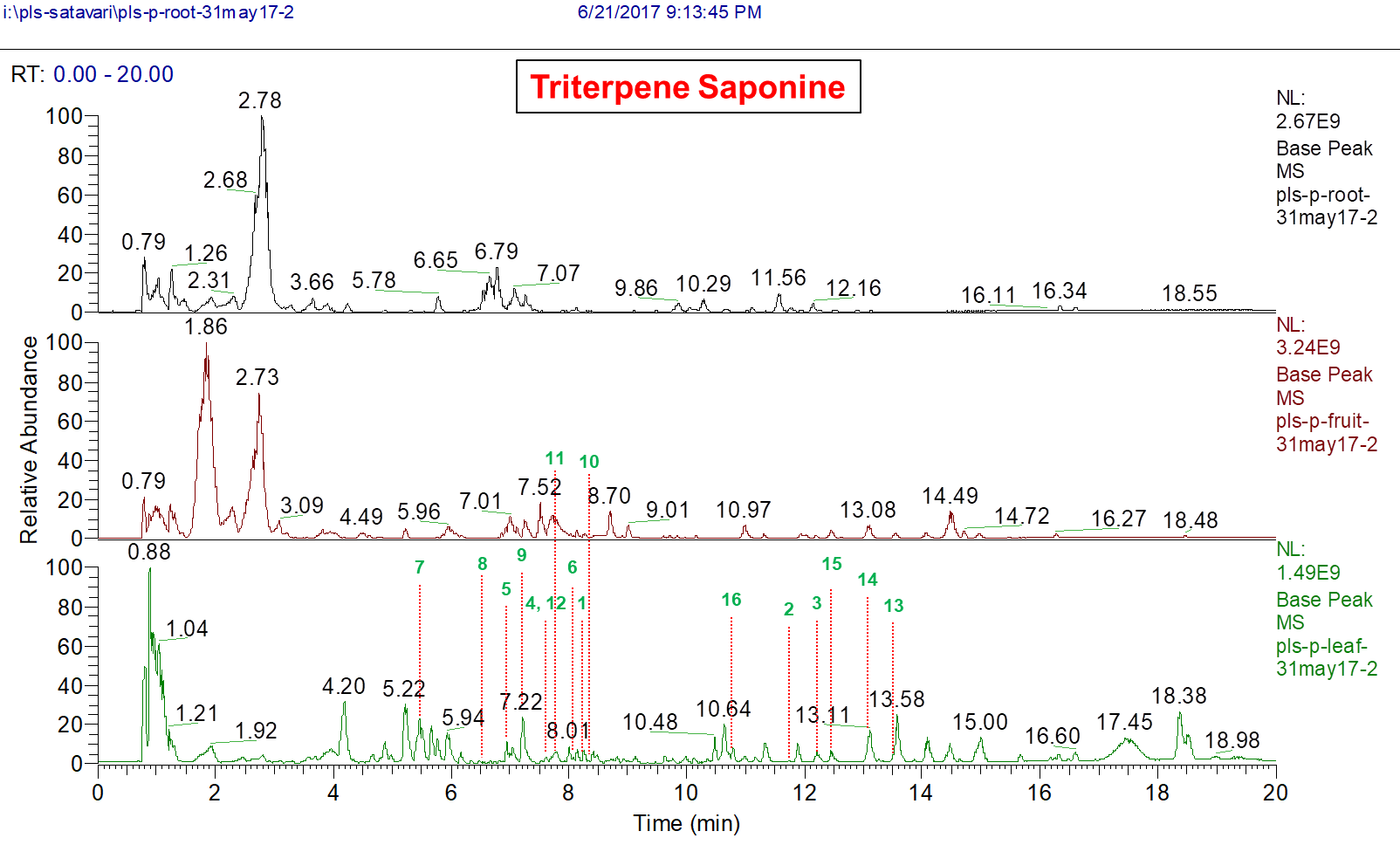


**Figure S14B:** LCMS profiles of extracts of root, leaf and fruit tissues of *A. racemosus* highlighting triterpene saponins.


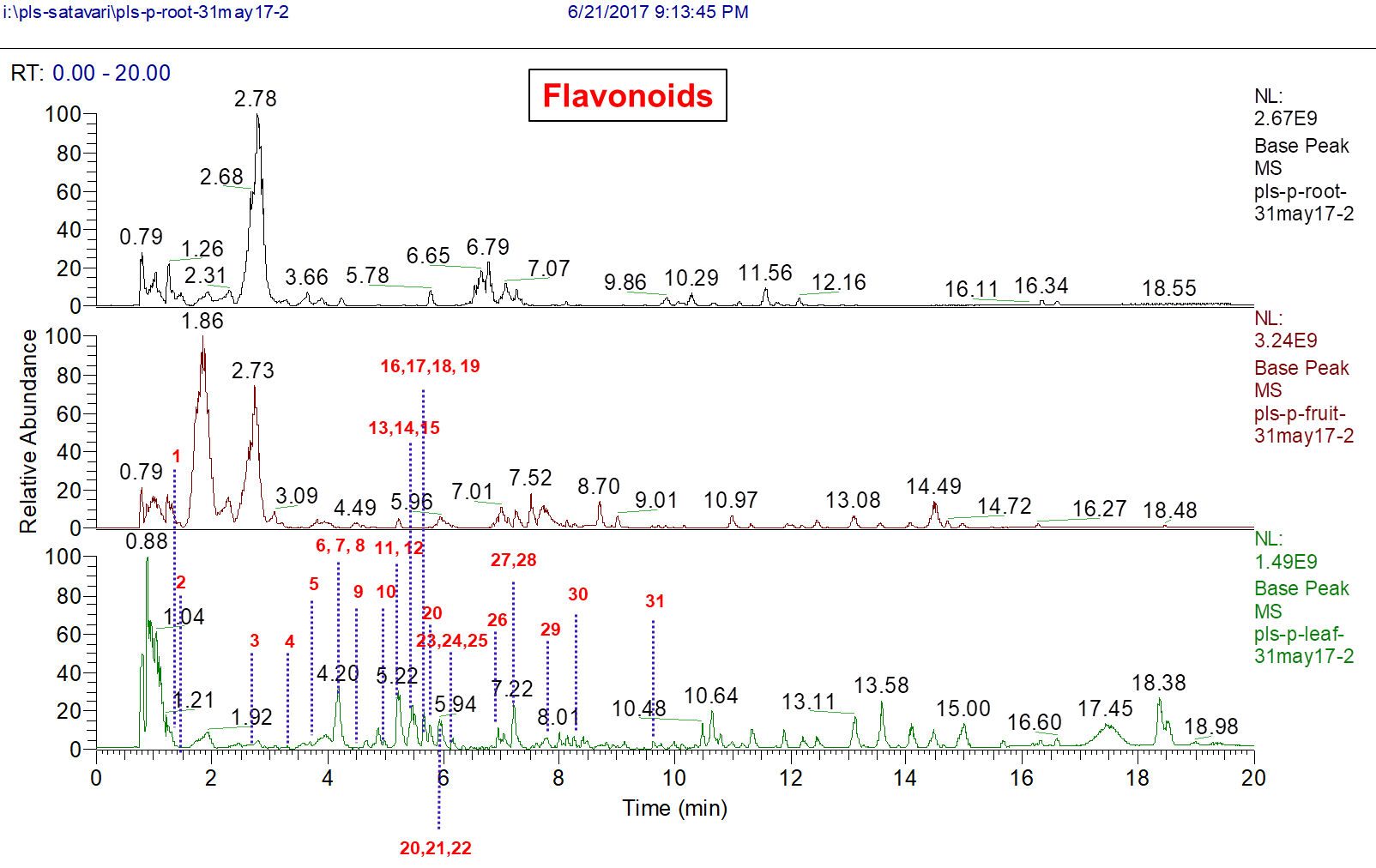


**Figure S14C:** LCMS profiles of extracts of root, leaf and fruit tissues of *A. racemosus* highlighting flavonoids.

**Figure S15:** Top 20 organisms showing significant match with *A. racemosus* transcriptome in similarity search with the available transcriptomics/genomics resource of plants reported till date.

**Figure S16:** KASS analysis.

**Figure S17:**  Transcripts specifically involved in secondary metabolite biosynthetic pathway.


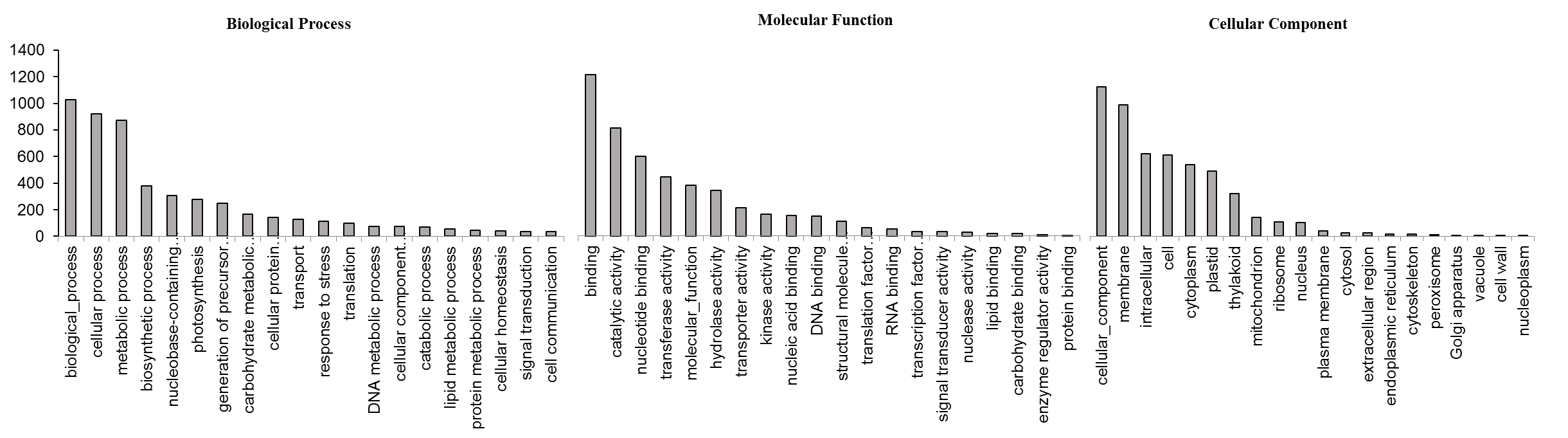


**Figure S18:** GO annotation of transcripts up-regulated in leaf as compared to root tissues.


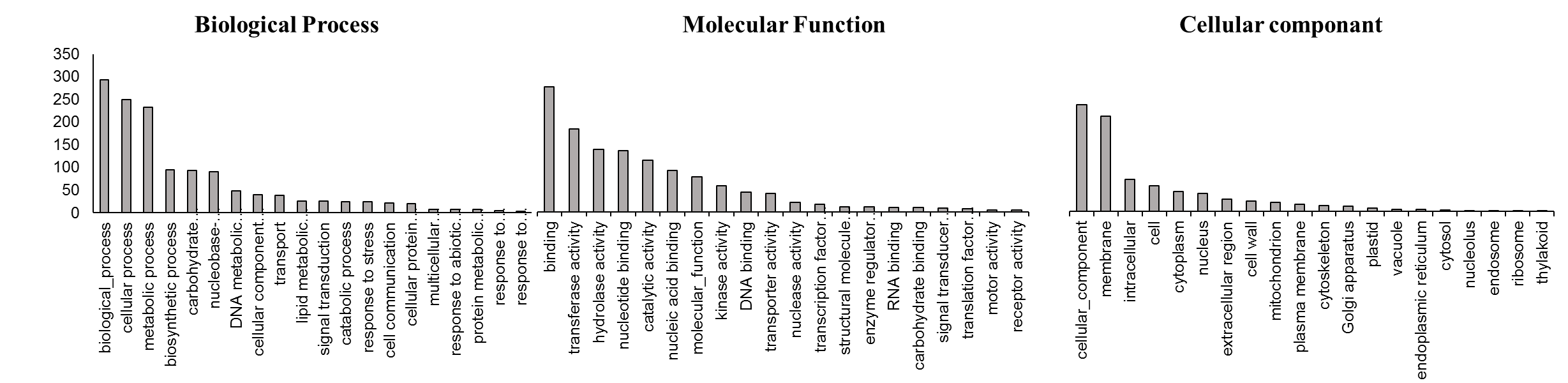


**Figure S19:** GO annotation of transcripts down-regulated in leaf as compared to root tissues.


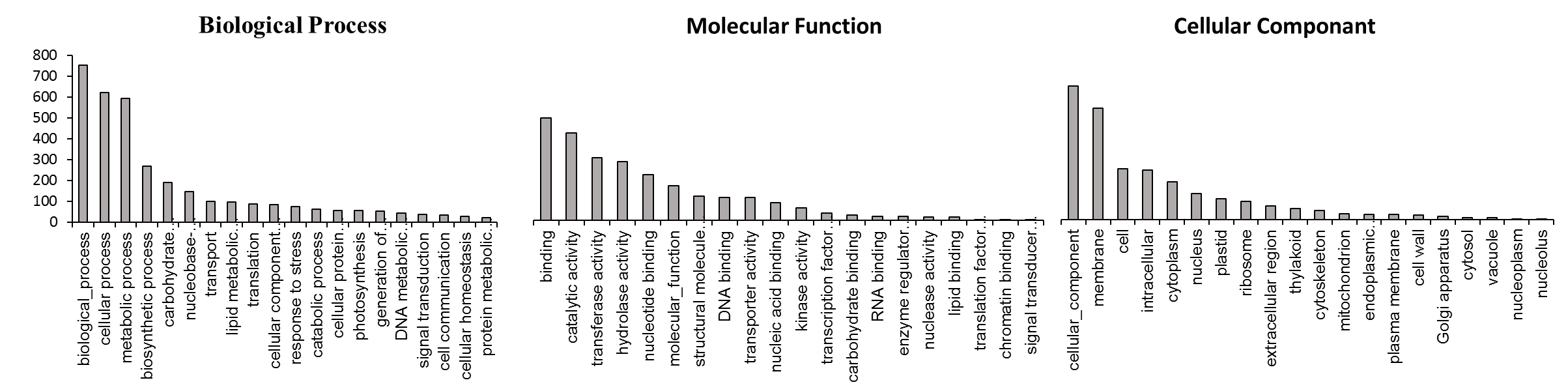


**Figure S20:** GO annotation of transcripts up-regulated in fruit as compared to root tissues.


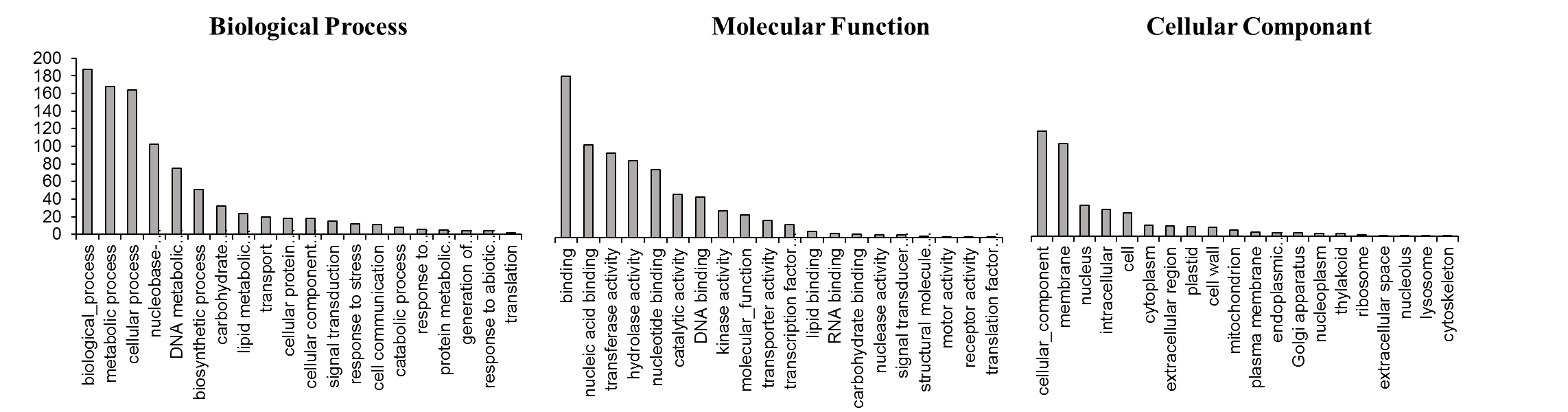


**Figure S21:** GO annotation of transcripts down-regulated in fruit as compared to root tissues.


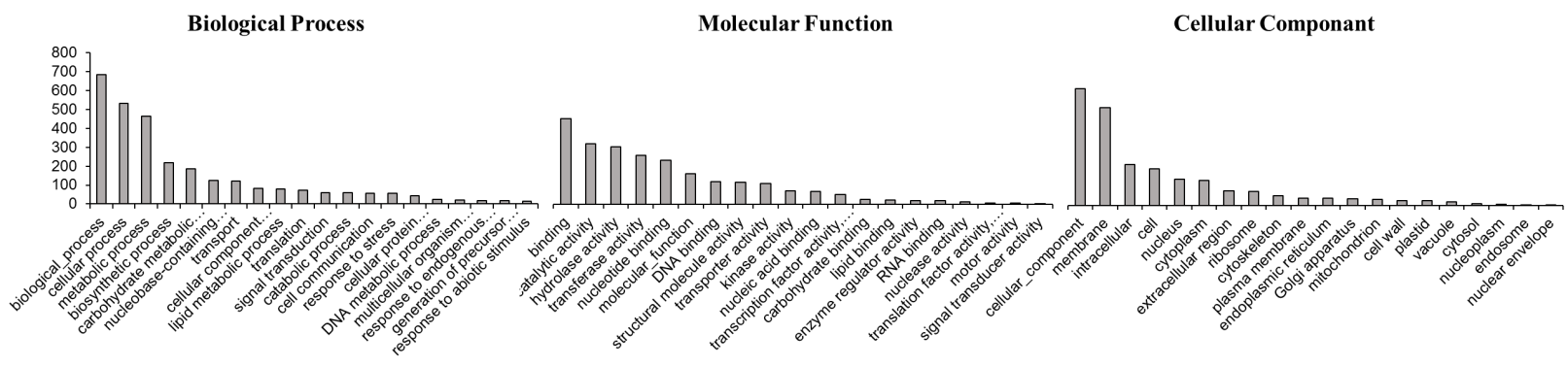


**Figure S22:** GO annotation of transcripts up-regulated in fruit as compared to root tissues.


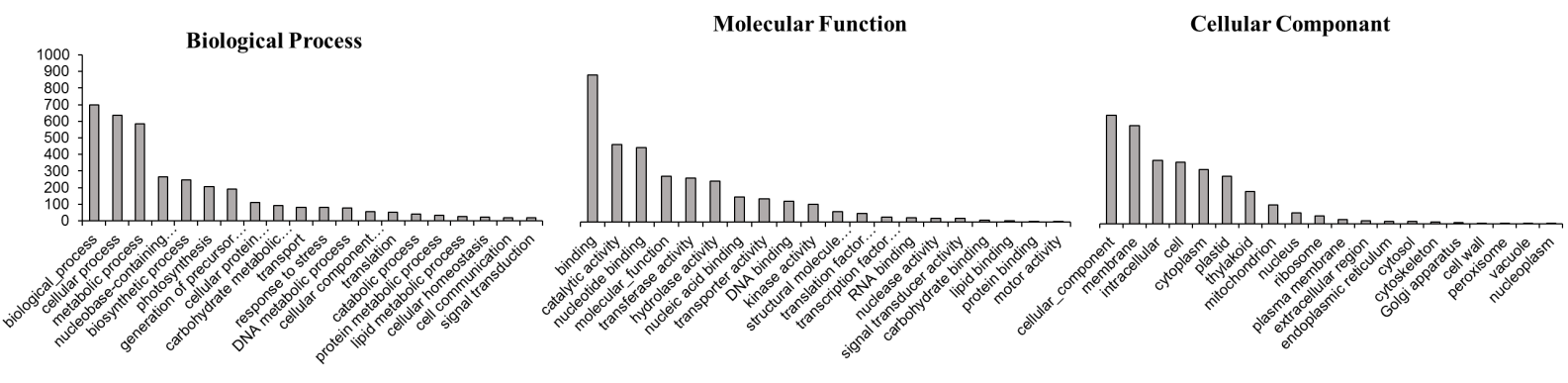


**Figure S23:** GO annotation of transcripts down-regulated in fruit as compared to root tissues.


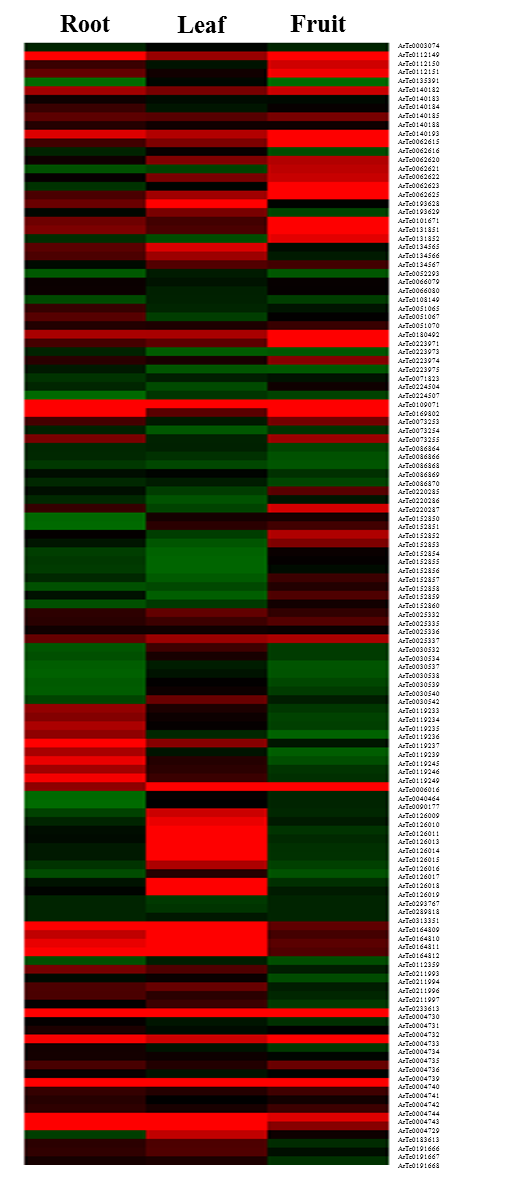


**Figure S24:** Differential gene expression pattern of MVA and MEP pathway genes in various tissues of *A. racemosus* transcriptome.


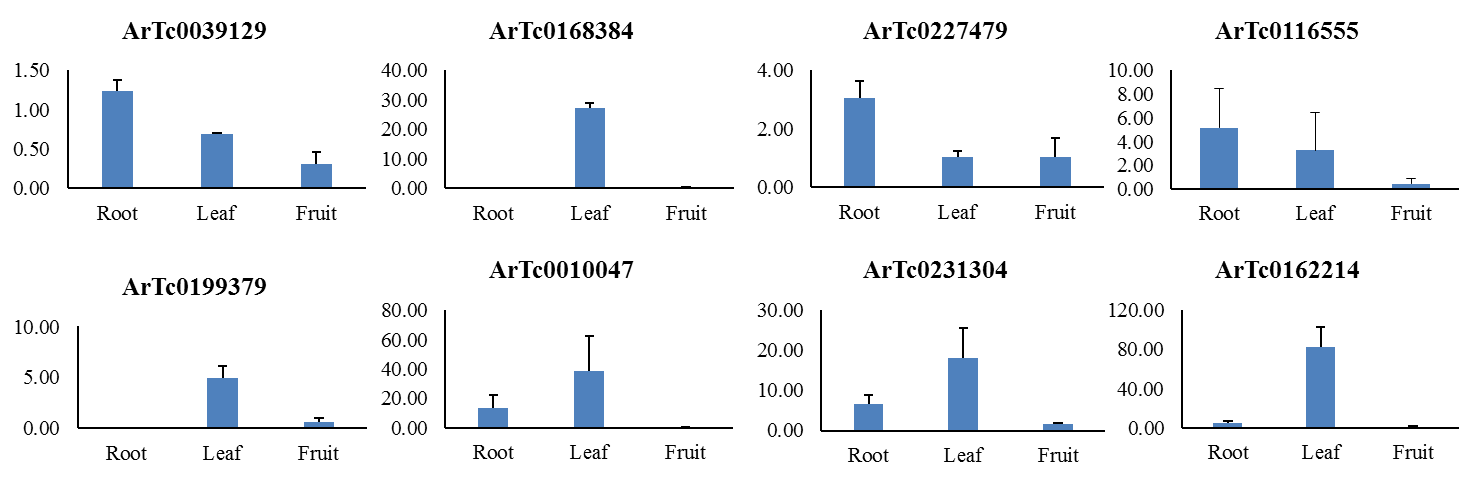


**Figure S25:** Real-time PCR validation for gene expression of 8 selected transcripts obtained from *A. racemosus* RNA-seq, ArTc0039129 (trans-ocimene synthase), ArTc0168384 (geraniol 8-hydroxylase), ArTc0227479 (cycloartenol synthase), ArTc0116555 (beta-amyrin hydroxylase), ArTc0199379 (chalcone synthase), ArTc0010047 (WRKY transcription factor), ArTc0231304 (sterol glucosyltransferase) and ArTc0162214 (flavonoid glucosyltransferase).


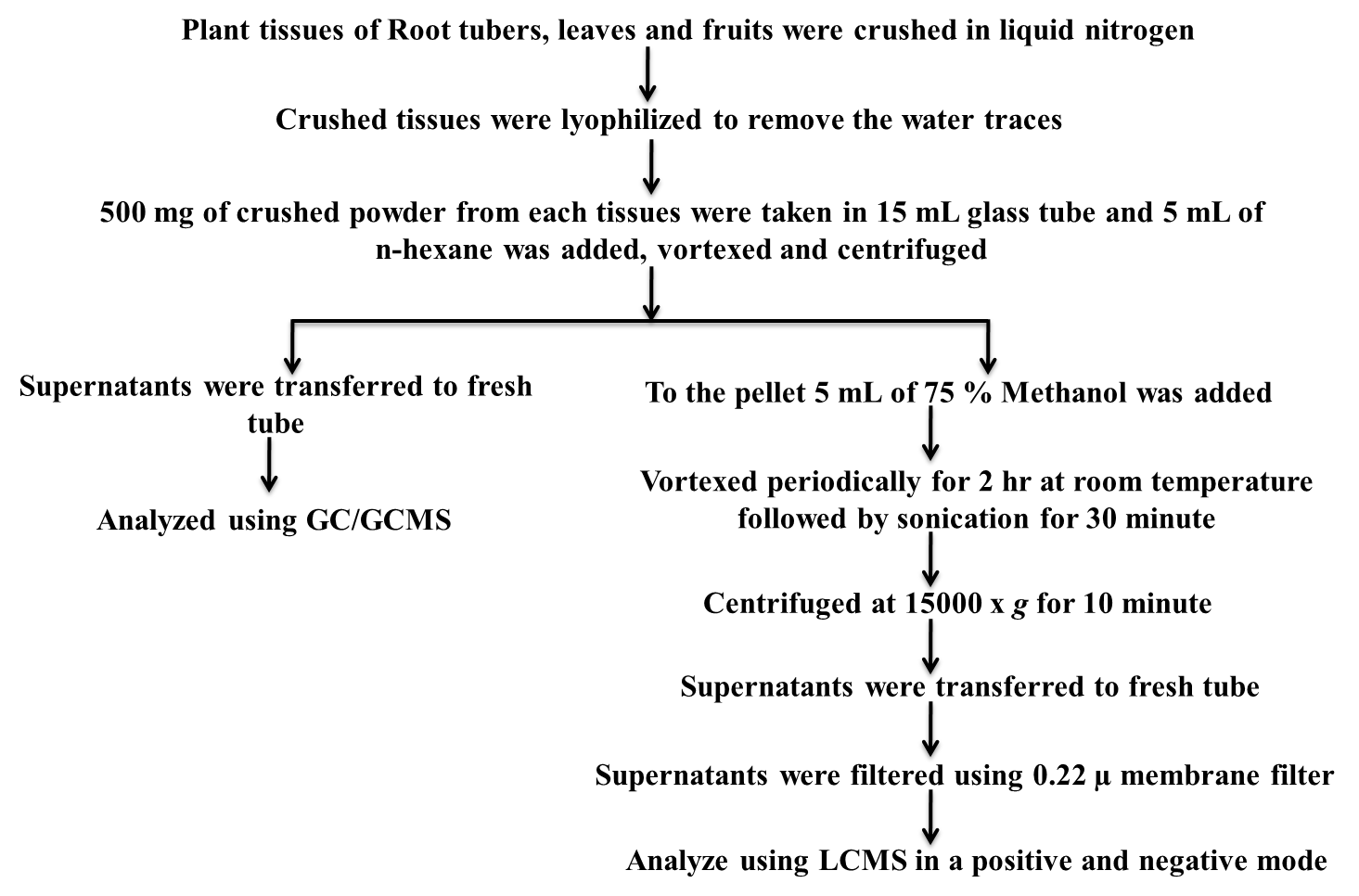


**Figure S26:** Schematic representation of metabolic extraction from various tissues (root, leaf and fruit) of *A. racemosus*.
